# Spatial positioning of EB family proteins at microtubule tips involves distinct nucleotide-dependent binding properties

**DOI:** 10.1242/jcs.219550

**Published:** 2018-10-31

**Authors:** Daniel Roth, Benjamin P. Fitton, Nikola P. Chmel, Natalia Wasiluk, Anne Straube

**Affiliations:** 1 Centre for Mechanochemical Cell Biology (CMCB), University of Warwick, Coventry CV4 7AL, UK; 2 Division of Biomedical Sciences, Warwick Medical School, University of Warwick, Coventry CV4 7AL, UK; 3 Molecular Organisation and Assembly in Cells (MOAC) Doctoral Training Centre, University of Warwick, Coventry CV4 7AL, UK; 4 Department of Chemistry, University of Warwick, Coventry CV4 7AL, UK

**Keywords:** Microtubules, Tip tracking, End-binding proteins, Tubulin, Nucleotide state, MAPs

## Abstract

EB proteins track the ends of growing microtubules and regulate microtubule dynamics both directly and by acting as the hub of the tip-tracking network. Mammalian cells express cell type-specific combinations of three EB proteins with different cellular roles. Here, we reconstitute EB1, EB2 and EB3 tip tracking *in vitro*. We find that all three EBs show rapid exchange at the microtubule tip and that their signal correlates to the microtubule assembly rate. However, the three signals differ in their maxima and position from the microtubule tip. Using microtubules built with nucleotide analogues and site-directed mutagenesis, we show that EB2 prefers binding to microtubule lattices containing a 1:1 mixture of different nucleotides and its distinct binding specificity is conferred by amino acid substitutions at the right-hand-side interface of the EB microtubule-binding domain with tubulin. Our data are consistent with the model that all three EB paralogues sense the nucleotide state of both β-tubulins flanking their binding site. Their different profile of preferred binding sites contributes to occupying spatially distinct domains at the temporally evolving microtubule tip structure.

## Abstract (precis)

**Summary:** *In vitro* reconstitution of tip tracking with EB1, EB2 and EB3 shows that these three proteins sense the nucleotide state of both β-tubulins flanking their binding site.

## INTRODUCTION

Microtubules are dynamic polymers that serve as structural elements and long-distance transport tracks in all eukaryotic cells. In addition, microtubule assembly and disassembly can be coupled to generate pushing and pulling forces. These functions of microtubules are essential for polarised cell growth and the faithful segregation of cellular contents during cell division. Microtubule assembly and disassembly is therefore tightly regulated by microtubule-associated proteins (MAPs) that either bind along microtubules or accumulate at their ends (van der Vaart et al., 2009). In particular, the localisation at the dynamic plus end of microtubules allows regulation of the assembly kinetics of microtubules and their interactions with structures inside the cell, such as kinetochores and the cell cortex (Montenegro Gouveia et al., 2010). Many microtubule regulators and motors depend on the highly conserved EB proteins for their accumulation at growing microtubule ends (Bieling et al., 2008; Dixit et al., 2009; Honnappa et al., 2009; Montenegro Gouveia et al., 2010; van der Vaart et al., 2011; Jiang et al., 2012; Duellberg et al., 2014; Thomas et al., 2016).

EB1, EB3 and their yeast homologues track the ends of growing microtubules autonomously (Bieling et al., 2007; Bieling et al., 2008; Komarova et al., 2009), and are thought to do so by recognising a nucleotide-dependent conformation of tubulin that is transiently formed during microtubule assembly. αβ-tubulin with GTP bound to the exchangeable site in β-tubulin is incorporated at the microtubule end. Addition of further subunits allows GTP hydrolysis and subsequent phosphate release. This results in GDP-tubulin forming the majority of the microtubule lattice. It is thought that exposure of GDP-tubulin at the microtubule end favours microtubule shrinkage, whereas a cap of GTP-tubulin stabilises the microtubule and allows polymer growth (Howard and Hyman, 2009). Use of slowly hydrolysable GTP analogues, such as GMPCPP or GTPγS, results in microtubules that are resistant to depolymerisation (Kirsch and Yarbrough, 1981; Hyman et al., 1992). Interestingly, these are also preferred substrates for EB binding (Zanic et al., 2009; Maurer et al., 2011), suggesting that plus-end tracking by EBs occurs via recognition of the nucleotide state of tubulin.

Whereas lower eukaryotes express only one EB protein (Beinhauer et al., 1997; Tirnauer et al., 1999; Rehberg and Gräf, 2002; Straube et al., 2003), mammalian cells have three paralogues: EB1 (also known as Mapre1) is ubiquitously expressed, but EB2 (also known as Mapre2) and EB3 (also known as Mapre3) are differentially regulated (Nakagawa et al., 2000; Su and Qi, 2001; Straube and Merdes, 2007; Goldspink et al., 2013). All three EB proteins share an N-terminal calponin homology (CH) domain that mediates microtubule binding (Hayashi and Ikura, 2003), an EB homology domain that mediates dimerisation and binding to +TIP proteins that contain an SxIP motif (Bu and Su, 2003; Honnappa et al., 2005, 2009), and a tubulin-like EEY motif at the C-terminus for binding to CAP-Gly proteins (Weisbrich et al., 2007). Interestingly, different cellular functions have been reported for EB1, EB2 and EB3: it has been noted that EB1 and EB3, but not EB2, are required for persistent growth of microtubules, the assembly of primary cilia and the recruitment of CLIP170 to microtubule ends (Komarova et al., 2005, 2009; Schroder et al., 2011). EB3 has a specific role in regulating the morphology of differentiating muscle and neuronal cells, the length of primary cilia and the stability of the midbody during cytokinesis (Straube and Merdes, 2007; Jaworski et al., 2009; Schroder et al., 2011; Ferreira et al., 2013). Mutations in EB2 cause craniofacial development defects and EB2 is involved in the regulation of cell adhesion and the reorganisation of microtubules in differentiating epithelia (Goldspink et al., 2013; Yue et al., 2014; Isrie et al., 2015). The molecular basis for these differential functions is largely unknown, and comprehensive studies into the different properties of the three EB paralogues are lacking.

Here, we explore differences in the microtubule-binding properties of EB1, EB2 and EB3. We find that EBs localise to spatially distinct sites at the microtubule end and reveal that EBs sense the nucleotide state of both β-tubulins adjoining their binding site. Amino acid changes in the microtubule interaction surface tune the binding affinities and preferences of the EB paralogues. This contributes to spatially distinct comet distributions when EBs compete for binding sites. Our study thus opens new investigations into how these differences in microtubule-binding properties contribute to the differential cellular functions of EB1, EB2 and EB3.

## RESULTS

### EB1, EB2 and EB3 track spatially distinct sites at microtubule ends in cells

**Fig. 1.**
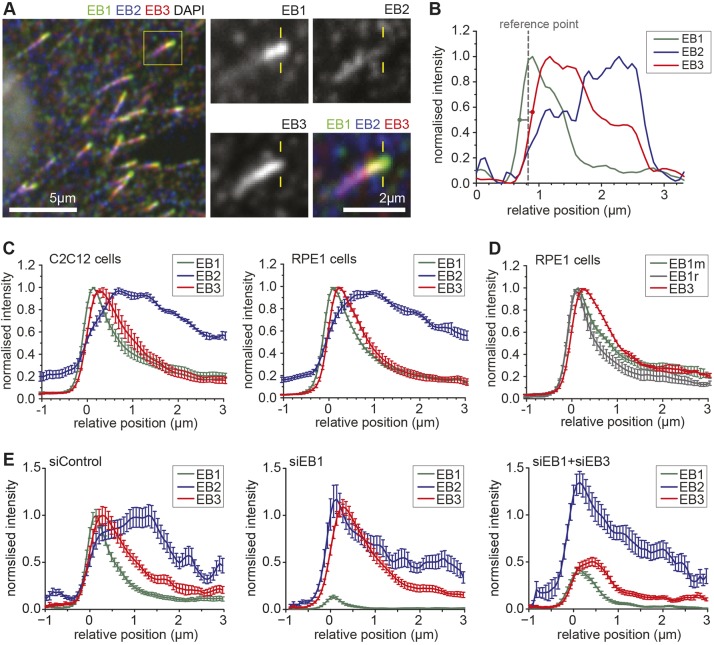
**EB1, EB2 and EB3 localise sequentially to the microtubule end.** (A) Immunolocalisation of EB1, EB2 and EB3 in C2C12 cells. (B) The line profile of EB1, EB2 and EB3 along the microtubule is derived from the inset in A. Microtubule plus end is on the left. Intensity values were normalised for each protein. The dashed grey line indicates the reference for alignment of line profiles across microtubules and experiments. (C) Averaged line profiles of EB1, EB2 and EB3 in C2C12 and RPE1 cells. Data from different microtubules were aligned at the midpoint between the first half-maximal values for EB1 and EB3 (position=0 µm), as indicated in B. Averaged values show data from 4–6 experiments using different combinations of fluorophores to exclude chromatic shift artefacts. *n*=56–104 microtubules from >5 cells per experiment. Error bars represent s.e.m. (D) Averaged line profiles of EB1 and EB3 in RPE1 cells. Endogenous EB1 was detected with mouse (green) or rat antibodies (grey). *n*=147–171 microtubules from four experiments with different fluorophore combinations as in C. Error bars represent s.e.m. (E) Averaged line profiles of EB1, EB2 and EB3 in RPE1 cells treated with siRNAs, as indicated, and averaged as data in C. *n*=28–47 microtubules. Error bars represent s.e.m.

To investigate the relative localisation of EB proteins in cells, we simultaneously stained endogenous EB1, EB2 and EB3 using specific antibodies in two unrelated mammalian cell lines, C2C12 murine myoblasts and RPE1 human retinal pigment epithelial cells. We observed that the three EB proteins did not colocalise (Fig. 1A). To quantitatively analyse, we obtained line profiles along the microtubule axis, and aligned these using the pixel closest to the mean location of the first half-maximum intensity values for EB1 and EB3 as a reference point (Fig. 1B), before averaging data from different microtubules. To exclude any effects caused by the different properties of fluorophores and any remaining chromatic aberration, we averaged data from experiments using different combinations of secondary antibodies (Fig. 1C). EB1 and EB3 show a similar shaped curve with a half-maximal width of ∼1 µm. We reproducibly find that the EB1 peak is located closer to the microtubule end than the EB3 peak, with a mean difference of 145 nm (*P*=8.8×10^−4^, paired Student's *t*-test). To exclude a difference caused by epitope masking in a subset of EB1, we confirmed the experiment using an alternative anti-EB1 antibody (Fig. 1D). EB2 localised to a several-micron-wide region with a broad peak 400–700 nm distal from EB3, consistent with previous findings (Komarova et al., 2009; Yang et al., 2017). We also confirmed previous findings that the EB2 peak shifts towards the tip when EB1 and EB3 are depleted (Komarova et al., 2009). Interestingly, EB2 levels increase in particular at the position of EB1, rather than that of EB3, when both EB1 and EB3 are depleted (Fig. 1E). Consistent with this, the efficient depletion of EB1 alone is sufficient to trigger the forward shift of EB2 (Fig. 1E). As the spatial positioning of EBs was identical across two unrelated cell lines and an understanding for the peculiar behaviour of EB2 is currently lacking, we investigated microtubule tip tracking of all three mammalian EB paralogues *in vitro*.

### EB1, EB2 and EB3 do not colocalise at microtubule ends *in vitro*

**Fig. 2.**
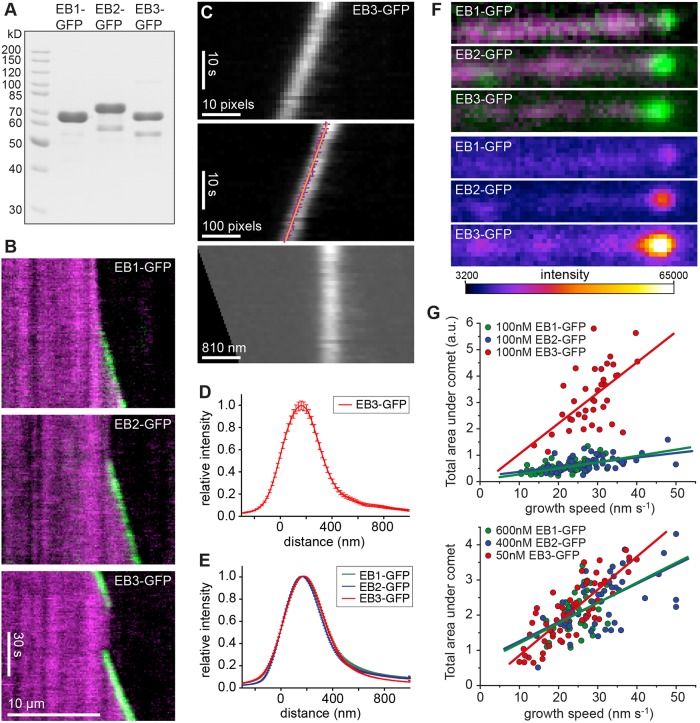
**EB1, EB2 and EB3 autonomously track the growing microtubule end.** (A) Coomassie Blue-stained polyacrylamide gel of purified EB1-GFP, EB2-GFP and EB3-GFP samples. (B) Kymographs showing tip tracking of 100 nM EB-GFP (green) on X-rhodamine microtubules (magenta). (C,D) Example of comet shape analysis using 100 nM EB3-GFP. Kymographs of linear growth phases were cropped and aligned using a linear fit through the peak values for each time point (C). The data were interpolated in space to allow shifts with a precision of 1/10 original pixel resolution. Data for each kymograph are summed in time (D). Error bars show s.e.m. (E) Comet shape data as in D were aligned at the first half-maximal point from 164–332 microtubules with a range of concentrations of EB1-GFP, EB2-GFP or EB3-GFP (see Materials and Methods for details). Error bars represent s.d. (F) Typical examples of 100 nM EB-GFP comets at growing microtubule ends, shown in green overlaid with microtubule (magenta) in top panels and with same intensity scaling according to the colour scale below. (G) EB-GFP protein binding as a function of microtubule growth speed. From the data used for E, the total area under the curve before normalisation was calculated and plotted relative to the growth speed for each growth phase analysed. The upper plot shows data for 100 nM of each EB-GFP, while the lower plot shows data for different amounts of EB-GFP that roughly correspond to physiological amounts of EBs, as found in undifferentiated C2C12 myoblasts judged by comparative western blots with serially diluted purified protein. a.u., arbitrary units.

To determine any intrinsic differences in microtubule binding, we purified recombinant GFP and mCherry fusion proteins of EB1, EB2 and EB3 (Fig. 2A) and added these to dynamic microtubules *in vitro*. Total internal reflection fluorescence (TIRF) microscopy showed that all three EB proteins autonomously track the growing plus end (Fig. 2B). Computing averaged intensity profiles from linear growth phases shows undistinguishable comet shapes for all three EBs (Fig. 2C–E). However, the total intensity of EB-GFP comets was dramatically different (Fig. 2F). At 100 nM EB-GFP, EB3 comets were 10× brighter than EB1 and 3× times brighter than EB2 [average tip intensities (×10^3^)±s.d.: EB1-GFP 4.9±1.9, EB2-GFP 14.8±4.5, EB3-GFP 47.3±4.7, *n*=116–162 microtubules]. All three EBs showed a linear correlation of instantaneous growth speed to total comet intensity (Fig. 2G) in agreement with data on Mal3 (Duellberg et al., 2016). Note that EB3 is more potent at increasing growth speed than EB1 [average instantaneous growth speeds±s.d.: control 9.3±7.4, 100 nM EB1-GFP 9.5±8.1, 100 nM EB2-GFP 13.0±8.8, 100 nM EB3-GFP 16.4±8.9, *n*=116–181 microtubules, each observed for 600 s at 1 frame per second (fps), all distributions are statistically different from control at level 0.05 or below in two-sample Kolmogorov–Smirnov test]. Therefore, increased comet intensity can be partially explained by faster assembly of microtubules in the presence of EB2 and EB3. Importantly, the data show that EB2 is an autonomous tip tracker, similar to other EBs investigated previously.

**Fig. 3.**
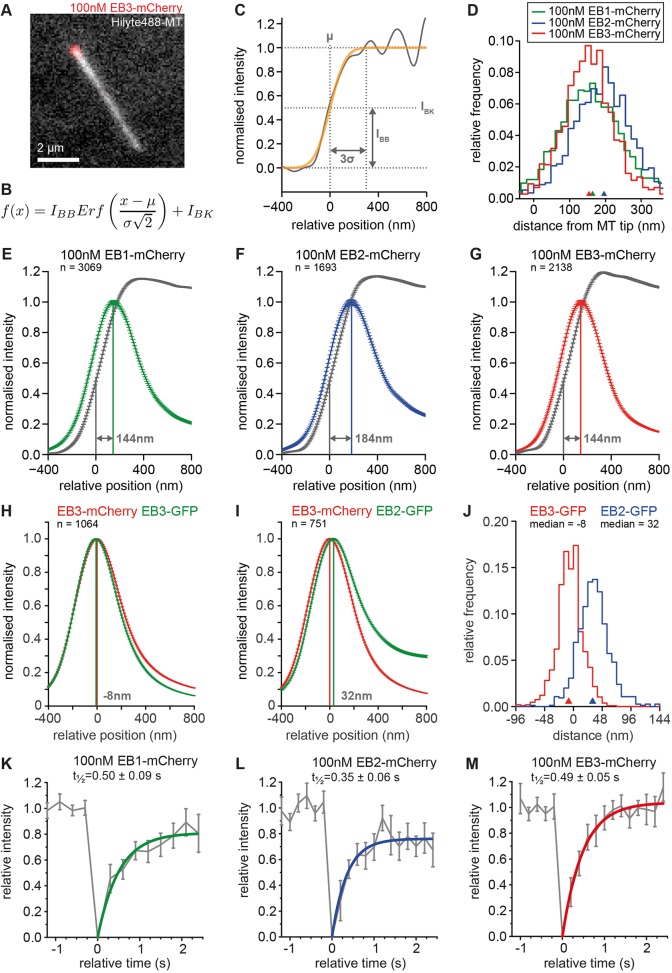
**EB1/EB3 and EB2 have distinct binding sites at microtubule ends *in vitro*.** (A) Example of a microtubule labelled with 17% Hilyte488-tubulin and 100 nM EB3-mCherry. (B,C) A Gauss error function (B, orange in C) was fitted to intensity data obtained along the length of the microtubule (grey) to determine the microtubule end position µ, which was set to 0 for alignment of curves from multiple microtubules. (D) Microtubules growing at a speed of between 10 and 30 nm/s and a Gauss error fit with a variance σ<200 nm were selected. The distance of EB peak to the microtubule tip is shown as a histogram. Triangles indicate the median. The EB1 distribution has been shifted by 1 pixel to improve clarity. Distributions are statistically different from each other (Kolmogorov–Smirnov test, *P*<10^−11^). *n*=1693–3069 measurements from 97–137 microtubules. (E–G) Intensity data from microtubules as in D were averaged relative to microtubule tip position for each EB. Error bars represent s.e.m. The distance between the microtubule tip and the EB peak is given. (H,I) Superaveraged intensity data from two-colour experiments with EB3-mCherry and EB-GFP as indicated. Peak distances are indicated in grey. (J) Histograms of peak distances for each microtubule growth phase analysed in the experiments shown in H and I. Distributions are statistically different from each other (Kolmogorov–Smirnov test, *P*<10^−158^). (K–M) Fluorescence recovery after photobleaching of EB signal at constantly growing microtubule ends. Averaged curves with exponential fit from 10–23 microtubules are shown for each protein. Error bars represent s.e.m.

To determine any differences in the position of the EB comets relative to the microtubule tip, we labelled microtubules with HiLyte488-tubulin and fitted a Gauss error function to the microtubule end to ascertain the position of the microtubule end with subpixel precision (Fig. 3A–C). We identified linear microtubule growth phases from the end-position data and selected those with a similar growth speed (10–30 nm/s) and a variance of the microtubule fit of less than 200 nm. These limitations were imposed to only compare blunt microtubules with a reliable curve fit at the end and to compare microtubules in a similar growth state. To estimate the accuracy of microtubule end detection in our experiments, we generated synthetic images of microtubules with a range of labelled tubulin densities and added experimental imaging noise. We then fitted the Gauss error function to each end of the microtubule and determined the difference between the measured microtubule lengths to the actual lengths of the simulated microtubules. This analysis shows that using 17% labelled tubulin and a signal-to-noise ratio of 6 to 11, as in our experiments, we can determine microtubule length accurately to ∼10 nm with a standard deviation of 50 nm (Fig. S1A). We then used two complementary approaches to determine the position of the EB peaks relative to the microtubule end. First, we averaged the intensity data using the position µ of the microtubule tip as a reference point. Determining the peak position of each EB in the averaged distribution gave results of 144 nm for EB1 and EB3 and 184 nm for EB2 (Fig. 3E–G). Applying the intensity averaging method for all analysed image stacks separately resulted in distributions for EB1, EB2 and EB3, with a median at the same position as the pooled data in Fig. 3E–G and standard deviations of 22–35 nm (Fig. S1B). A Kolmogorov–Smirnov test on these distributions suggested that the distributions of EB1 and EB3 are not significantly different (*P*=0.798), while the EB2 distribution is different from both EB1 and EB3 distributions (*P*=0.0180 and 0.0184, respectively). As an independent second approach, we fitted for each data point a Gaussian to the peak of the EB signal and determined the distance to the microtubule tip position. The median peak positions were 164 nm for EB1, 196 nm for EB2 and 155 nm for EB3 (Fig. 3D, distributions are significantly different from each other).

In cells, the EB paralogues are present simultaneously, thereby excluding the possibility that differences in the tip structure or nucleotide composition results in altered peak positions. To test whether we can recapitulate spatially distinct binding *in vitro* with mixed EBs, we performed experiments adding both EB3-mCherry and EB2-GFP at the same time and determining their relative comet positions. As a control, EB3-mCherry and EB3-GFP were used. We found that EB3-GFP was ∼8 nm closer to the microtubule tip than EB3-mCherry, but EB2-GFP was 32 nm behind (Fig. 3H–J). The distributions are significantly different from each other (*P*=2.5×10^−159^, Kolmogorov–Smirnov test). These results confirm that the more distal binding of EB2 from the microtubule tip we observed in cells (Fig. 1C) can be reproduced *in vitro*, albeit with a smaller magnitude.

### EB1, EB2 and EB3 have different preferences for the nucleotide state of tubulin in the microtubule lattice

EB1 and EB3 tip track by recognising and rapidly exchanging at a nucleotide-dependent binding site that is transiently formed at growing microtubule ends (Zanic et al., 2009; Maurer et al., 2011; Montenegro Gouveia et al., 2010). One possible explanation for the distally shifted localisation of EB2 is that its landing rate and binding duration at the microtubule end might be different. We performed fluorescence recovery of photobleaching experiments to detect any such differences in protein turnover at the microtubule end. In agreement with the literature (Bieling et al., 2008; Montenegro Gouveia et al., 2010), EB1 and EB3 exchange is in the subsecond range. We find that EB2 turns over 30% faster than EB1 and EB3 (Fig. 3K–M). Thus, all three EBs undergo several cycles of unbinding and rebinding during the lifetime of their binding site at a particular location in the assembling microtubule tip, and our data exclude a kinetic model whereby delayed binding and release would result in a more distal position of EB2 at the microtubule tip.

**Fig. 4.**
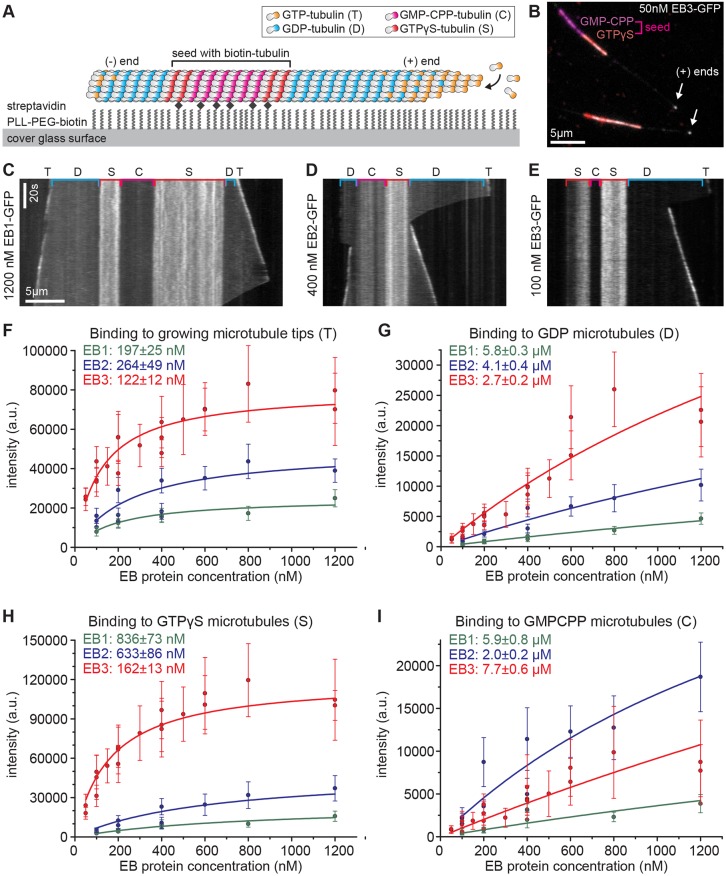
**EB proteins have different nucleotide preferences.** (A) TIRF-based microtubule-binding assay using dual-labelled seeds stabilised with GMPCPP and GTPγS, respectively. Dynamic microtubule extensions were unlabelled. (B) Example image of 50 nM EB3-GFP (greyscale) on different microtubule-binding sites. (C–E) Example kymographs from timelapse images. Note that different concentrations of EB1-GFP, EB2-GFP and EB3-GFP have been selected that show comparable plus-tip labelling. Different substrates are indicated with single-letter codes as in A. (F–I) Binding curves for EB-GFPs on four different microtubule substrates measured as fluorescence intensity from timelapse images. Data points represent mean±s.d. from >25 microtubules each; data from different experiments are plotted as separate data points. Tip-binding curves were fitted with I=I_max_•[EB]/(K_D_+[EB]) and thereby determined I_max_ values (25,000 for EB1, 50,000 for EB2 and 80,000 for EB3) were fixed for curve fits in G–I, except for EB3 in H for which 120,000 was used. Fitted values for K_D_ are provided in the key for each graph.

We next explored the possibility that EB2 prefers different binding sites from EB1 and EB3. Based on the observation that EB1 and its *Schizosaccharomyces* *pombe* orthologue Mal3 preferentially bind to microtubules made with tubulin bound to the GTP analogues guanosine-5′-[(α,β)-methyleno]triphosphate (GMPCPP) and guanosine-5′-(γ-thio)-triphosphate (GTPγS), the EB binding site is thought to be determined by the nucleotide state of tubulin (Zanic et al., 2009; Maurer et al., 2011, 2012). To determine whether the three mammalian EBs have different preferences for the nucleotide state of tubulin, we measured their binding to microtubule-containing regions with different nucleotides. We made GMPCPP-stabilised microtubules, elongated these with GTPγS-tubulin and used these as seeds in a plus-end-tracking assay in the presence of 12 µM GTP-tubulin (Fig. 4A,B). TIRF microscopy allowed the simultaneous detection of EBs binding to four different substrates – microtubule lattices with GMPCPP-, GTPγS- or GDP-tubulin and growing microtubule tips containing a mosaic of GTP- and GDP-tubulin – plus potential intermediates such as GDP/P_i_-bound tubulin (Fig. 4A–E). EB3 has the highest affinity as well as the highest density of binding sites at the microtubule tip, the GDP lattice and GTPγS microtubules (Fig. 4F–H). This is consistent with data from cells expressing different levels of EB-GFP, in which the tip intensity was measured versus the cytoplasmic background intensity (Fig. S2). However, on GMPCPP microtubules, EB2 has the highest affinity and is the only EB protein that prefers GMPCPP-tubulin over GDP-tubulin under these experimental conditions (Fig. 4I). Although all three EB paralogues prefer GTPγS microtubules, our data suggest that EB2 might additionally bind to a slightly different conformation of tubulin that is present in GMPCPP microtubules.

### EBs recognise the nucleotide state of both β-tubulins adjoining their binding site

**Fig. 5.**
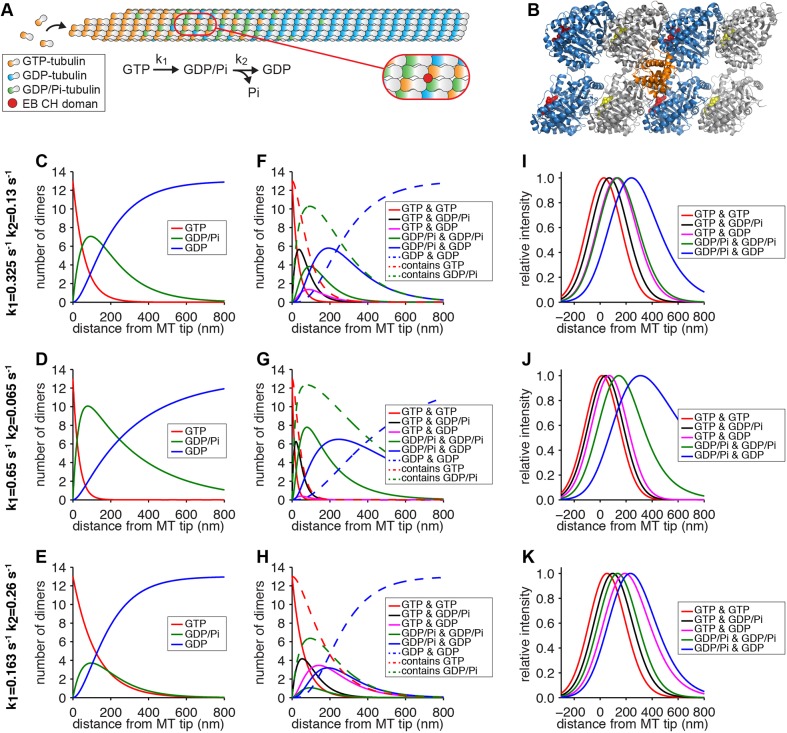
**Simulation of paired nucleotide distributions.** (A) Schematic representation of nucleotide distribution at the growing microtubule end, assuming uncoupled, first-order kinetics of GTP hydrolysis (at rate k_1_) and phosphate release (at rate k_2_). Zoomed section shows the binding site for the CH domain of EB proteins at the interface of four tubulin subunits. (B) Atomic model of EB3 CH domains (orange) binding a GTPγS microtubule lattice (Protein Data Bank 3JAK). α-tubulin is shown in grey, β-tubulin in blue, GTP in the nonexchangeable site in yellow and GTPγS in the exchangeable site in red. Note the proximity of EB3 to two exchangeable nucleotide sites. (C–E) Distribution of GTP-tubulin, GTP/P_i_-tubulin and GDP-tubulin relative to the microtubule end, assuming uncoupled, first-order kinetics of GTP hydrolysis and phosphate release for three combinations of reaction rates as indicated. Rates used are based on measurements by Melki et al. (1996) (C) plus variations of 2-fold different rates (D,E) and calculated for 20 nm/s growth. (F–H) Distribution of nucleotide combinations bound to neighbouring tubulin dimers derived from distributions in C–E. (I–K) Distributions from F–H after normalisation and convolution with a Gaussian to approximate the experimentally obtained point-spread function for GFP.

To further explore the hypothesis that EB proteins could bind to different nucleotide-dependent binding sites on the microtubule tip, we next simulated the distribution of tubulin in different nucleotide states at the microtubule end. High-resolution structures of GTPγS microtubules show that the Mal3 and EB3 CH domains bind at the interface of four tubulin subunits (Maurer et al., 2012; Zhang et al., 2015). Thus, an EB protein might be able to detect the nucleotide state of both β-tubulins adjoining its microtubule-binding site (Fig. 5A,B). Tubulin subunits are incorporated at the microtubule tip when β-tubulin is bound to GTP. GTP hydrolysis and phosphate release are triggered after incorporation into the microtubule lattice. For our simulations, we assume two reactions with first-order kinetics: GTP hydrolysis, GTP → GDP/P_i_, with rate constant k_1_; and phosphate-release, GDP/P_i_ → GDP+P_i_ with rate constant k_2_ (Fig. 5A). Both rates have previously been determined experimentally for microtubules assembled in the presence of Taxol at 25°C, with k_1_ in the range of 0.3–0.35 s^−1^ and k_2_ in the range of 0.11–0.15 s^−1^ (Melki et al., 1996). As these values might deviate under conditions that permit dynamic instability, we also tested combinations of 2-fold higher and lower rates for our simulations. We first calculated the distribution of three different nucleotide states – GTP, GDP/P_i_ and GDP – as a function of the distance from the microtubule tip for an average growth rate of 20 nm/s as in our experiments (Fig. 5C–E). Based on these distributions, we determined the probability of finding certain combinations of nucleotides in laterally adjoining tubulin dimers and obtained a number of comet-shaped distributions shifted by several tubulin layers (Fig. 5F–H). To illustrate how these distributions would show as GFP intensity data from a TIRF experiment, we normalised and convolved the data with the experimentally determined point-spread function of our TIRF setup. The result is a series of very similar, comet-shaped curves distributed along the microtubule (Fig. 5I–K), which closely resemble those obtained in our EB localisation experiments (Figs 2 and 3). The ratio of k_1_ and k_2_ determines the offset, decay and also the sequential order of certain distributions (Fig. 5I–K).

**Fig. 6.**
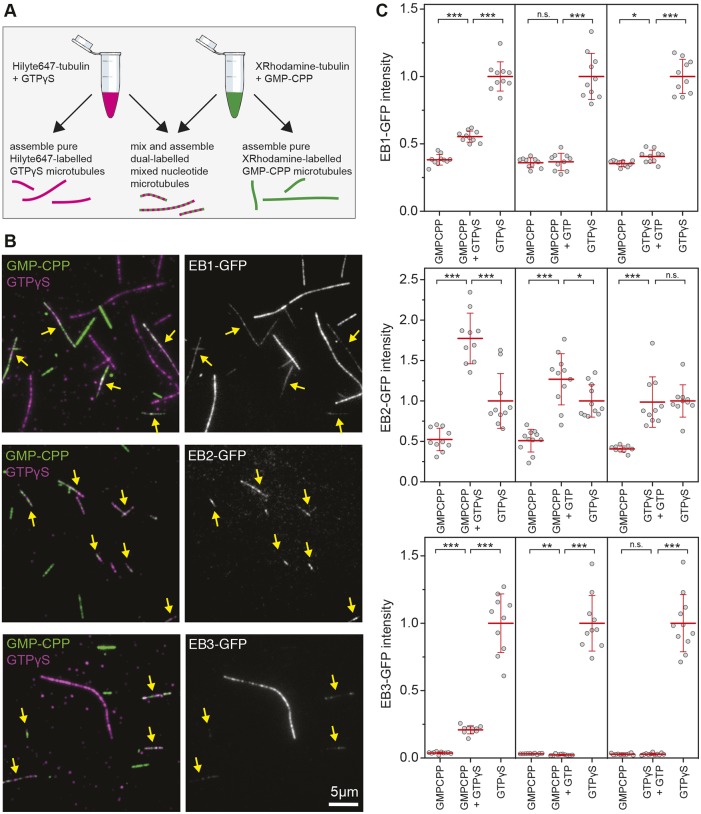
**Mixed-lattice binding.** (A) Schematic representation of a mixed-lattice experiment in which microtubules are co-assembled from tubulin pre-equilibrated with either GMPCPP or GTPγS. See Figs S3 and S4 for validation of proportional nucleotide incorporation. (B) Examples of mixed-lattice binding experiments with pure GMPCPP (green), pure GTPγS (magenta) or 1:1 mixed GMPCPP and GTPγS microtubules (indicated by yellow arrows) with EB-GFPs as indicated. Note the preference of EB2 for mixed lattice microtubules. (C) EB-GFP intensity measurements from mixed-lattice binding using microtubules assembled with 1:1 mixtures of tubulin bound to GMPCPP, GTPγS or GTP. Each data point represents a measurement for one field of view with 3–20 microtubules of each type. Data have been normalised so that the mean value of pure GTPγS microtubules is 1 for each experiment. Red lines indicate mean±s.d. n.s., nonsignificant; *P<0.05, **P<0.005, ***P<0.0005 (Student's *t*-test).

These theoretical distributions would explain both the small positional shift along the microtubule while retaining a similar comet shape, and also the apparent different saturation levels for the different EBs, as not all nucleotide combinations are equally abundant. To test whether EBs are indeed able to recognise the nucleotide state of two adjoining tubulins, we first tested whether one of the EBs might prefer a microtubule lattice with mixed nucleotide states. To do this, we equilibrated tubulin with different nucleotides before mixing these 1:1 immediately before warming the solution for assembly. To confirm whether mixed incorporation to the microtubule occurred, we added differently fluorescently labelled tubulin to each equilibration mixture (Fig. 6A). Using this technique, we successfully assembled microtubule lattices containing GMPCPP and GTPγS, GMPCPP and GDP (assembled as GTP), GTPγS and GDP, in addition to pure GMPCPP and GTPγS-containing lattices (Fig. 6). As the assembly kinetics of GMPCPP-tubulin is very rapid whereas assembly of GTPγS-tubulin is very slow, we analysed both the relative incorporation of fluorescently labelled tubulin (Fig. S3) and the nucleotide composition of the mixed microtubule lattices obtained from co-assembling GMPCPP-tubulin and GTPγS-tubulin using perchloric acid (PCA) extraction and high-performance liquid chromatography (HPLC) analysis (Fig. S4). Both analyses support the idea that incorporation of GMPCPP-tubulin and GTPγS-tubulin is equally efficient during co-assembly, and that they form mixed lattice microtubules with proportional nucleotide composition.

The different fluorescent labels allowed the side-by-side comparison of EB binding to both pure and mixed substrates in a single imaging chamber. We found that EB1 and EB3 preferred pure GTPγS microtubules, and reducing GTPγS-tubulin content reduced EB1 and EB3 binding dramatically (Fig. 6B,C). However, in agreement with our hypothesis, we found a preference of EB2 for binding to lattices containing mixed nucleotides. When co-polymers of GMPCPP- and GTPγS-tubulin, as well as GMPCPP- and GDP-tubulin, were substrates, EB2 bound significantly better than pure GMPCPP or GTPγS microtubules (Fig. 6B,C).

**Fig. 7.**
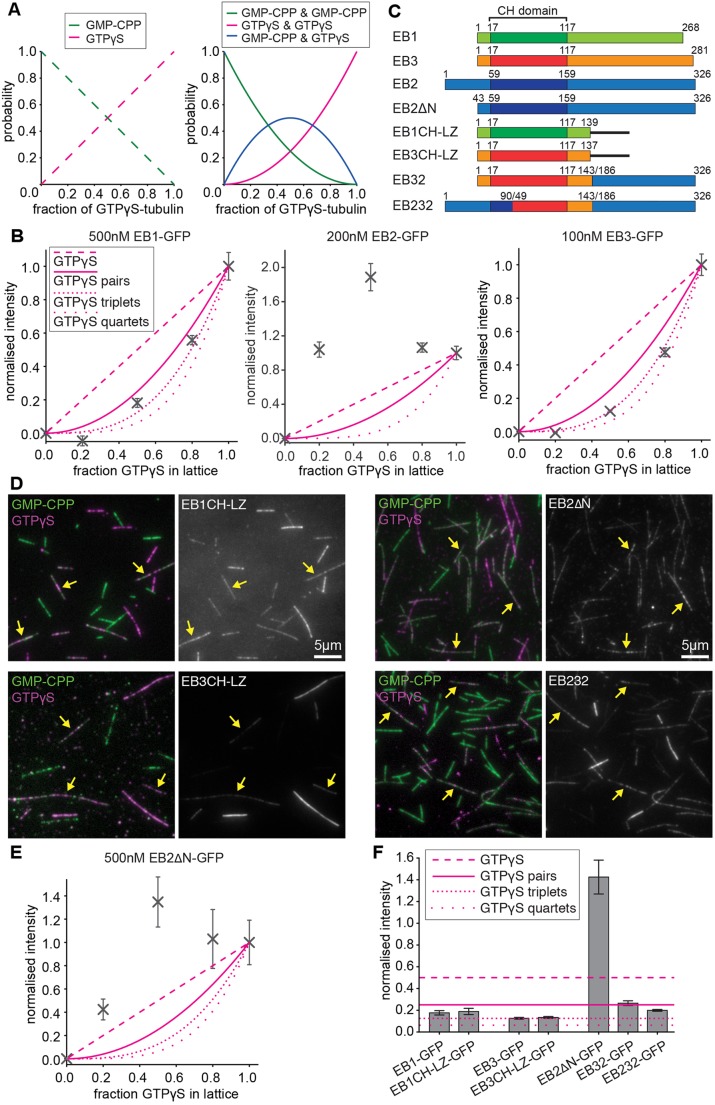
**The EB CH domain recognises the nucleotide state of tubulin pairs.** (A) Theoretical distribution of nucleotides and the probability of nucleotide pairs bound to neighbouring tubulin dimers in mixed-lattice experiments. (B) Relative intensity of EB-GFP bound to mixed lattices assembled from different relative amounts of tubulin pre-equilibrated with GMPCPP or GTPγS. Intensity was normalised to pure GMPCPP (set to 0) and pure GTPγS lattices (set to 1). Data show mean±s.e.m., *n*=10 fields, >50 microtubules. Theoretical density of GTPγS singles, pairs, triplets and quartets are indicated for comparison. (C) Schematic representation of EB constructs used in this figure. The microtubule-binding CH domain is highlighted in a darker shade. Constructs without the C-terminus contain a leucine zipper for dimerisation. EB2ΔN is truncated to mimic the EB1 and EB3 N-terminus. EB32 and EB232 are chimera of EB2 and EB3 as indicated. (D) Examples of mixed-lattice binding experiments with pure GMPCPP (green), pure GTPγS (magenta) or 1:1 mixed GMPCPP and GTPγS microtubules (indicated by yellow arrows) with EB-GFPs as indicated. (E) Relative intensity of EB2ΔN-GFP bound to mixed lattices as in B and C. (F) Relative intensity of EB-GFP constructs bound to mixed lattices assembled and analysed as in B and C. Theoretical density of GTPγS singles, pairs, triplets and quartets are indicated for comparison.

Next, we aimed to understand whether EB1 and EB3 recognise the nucleotide state of two adjoining tubulins or whether their binding scales directly with the concentration of GTPγS in the microtubule lattice (Fig. 7A). If dual-nucleotide recognition occurs, we would expect that EB1 and EB3 intensities correlated to the squared concentration of GTPγS as this represents the probability of finding binding sites flanked by a pair of GTPγS-tubulin in the microtubule lattice (Fig. 7A). Indeed we find when examining the intensity on mixed lattices co-assembled with different ratios of GMPCPP and GTPγS that the distribution of EB1 and EB3 follows the distribution of GTPγS pairs or triplets rather than monitoring the GTPγS concentration directly, whereas the distribution of EB2 follows a bell-shaped curve that matches the theoretical distribution of mixed pairs of nucleotides in the lattice (Fig. 7A,B). These data support the idea that all three mammalian EB proteins recognise a binding site that is sensitive to the nucleotide state of two or more neighbouring tubulin subunits in the microtubule lattice.

### Structural determinants of tip-tracking specificity

EB2 is the most divergent of the three mammalian EB proteins; most notably it contains a 42-amino acid N-terminal extension. To test whether the unique N-terminus confers the difference in microtubule recognition, we made an EB2 construct with an analogous N-terminus to EB1 and EB3, EB2ΔN (Fig. 7C). However, in mixed-lattice experiments, the truncated EB2 still preferred mixed over pure microtubule lattices and showed a bell-shaped curve similar to EB2 (Fig. 7C–E).

We next asked whether the dual nucleotide recognition involves any regions outside the CH domains. To do this, we deleted the C-terminal dimerisation domain and most of the linker region from EB1 and EB3, and added a leucine zipper from yeast GCN4 transcription factor to retain the dimerisation status of the protein. Both CH domains were sufficient to recapitulate the nucleotide preference of full-length EB1 and EB3 (Fig. 7D,F). Likewise, a chimera including the CH domain of EB3 and the tail of EB2 behaved similarly to EB3 (Fig. 7B,F). We next generated chimera to test whether transferring part of the amino acid changes in the microtubule-binding interface of EB3 to EB2 is sufficient to result in an EB3-like binding preference. A chimera, EB232, that contained 20 amino acid substitutions compared with EB2 (Fig. 7C; Fig. S5) showed a binding preference very similar to EB3 (Fig. 7D,F). Of these 20 amino acid substitutions, only six residues are within a distance of 5 Å from a tubulin residue, according to the EB3-GTPγS microtubule structure from the Nogales laboratory (Zhang et al., 2015) (Fig. S5). Two of these residues might make contact with the C-terminal tails of α1 (Fig. S5) and are not conserved between EB1 and EB3. A cluster of four EB2-specific residues at the interface with the right-hand-side protofilament (in plus-end-up view) is conserved between EB1 and EB3 and thus likely to confer the different binding specificity between EB1/EB3 and EB2 (Fig. S5). These residues are exchanged to an amino acid with conserved charge that is slightly less bulky (I90V, F105L and E106D) or slightly more bulky (K100R) in EB2.

**Fig. 8.**
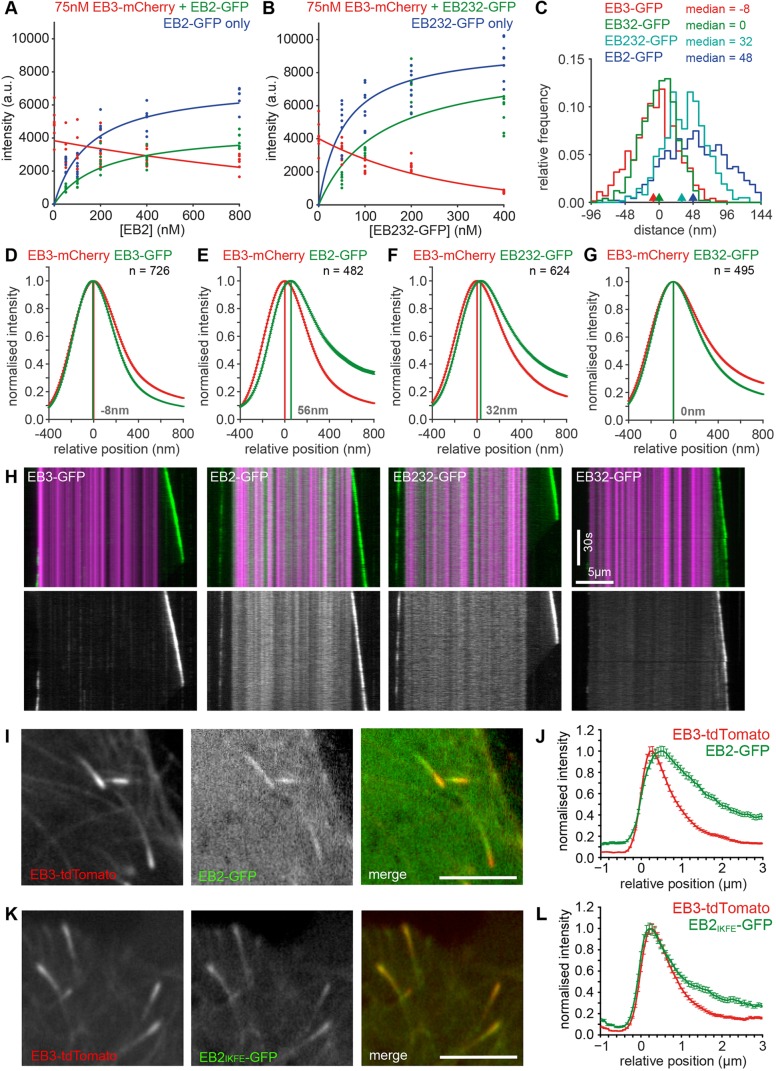
**Tip positioning and competition of EB chimera.** (A) Tip-binding intensities of EB2-GFP in the absence (blue) or presence (green) of 75 nM EB3-mCherry (red). *n*=7–8 fields of view with 10 microtubules each. Green and blue curves were fitted using a one-site-specific binding model; red curves were fitted using with an exponential decay function. Fit parameters EB2 only: K_D_=153± 117 nM, Bmax=7283±1916; EB2: K_D_=211±135 nM, Bmax=4496±1110; EB3: K=1471±947 nM. (B) Same as A, but using EB232-GFP. Fit parameters EB232 only: K_D_=56±39 nM, Bmax=9636±1876; EB232: K_D_=132±416 nM, Bmax= 8739±11,057. EB3: K=269±378 nM. (C) Histograms of peak distances for each microtubule growth phase analysed in the experiments shown in D–G. Arrowheads indicate medians. (D–G) Superaveraged intensity data from two-colour experiments with EB3-mCherry and EB-GFPs as indicated. Peak distances are shown in grey. (H) Representative kymographs showing GMPCPP-stabilised seeds (magenta) and EB-GFP (green) plus EB3-GFP as a greyscale image. Note the higher seed/GDP-lattice ratio for EB2 and EB232. (I,K) Simultaneously acquired images of the RPE1 EB3-tdTomato cell line expressing EB2-GFP or EB_IKFE_-GFP. Scale bars: 5 µm. (J,L) Line scans of growing comets, aligned to the first half-maximal signal of EB3, averaged and normalised to peak intensity. Data show mean±s.e.m. from 138–151 microtubules, 33 cells and 3–4 experiments.

We next asked how these amino acid changes affect EB232 tip tracking. We found that the EB232 chimera showed a 3-fold increased affinity for the microtubule tip and was able to compete with EB3-mCherry more efficiently than EB2 (Fig. 8A,B). EB232 also had a significantly reduced peak distance to EB3 (Fig. 8C–F). Importantly, adjusting protein levels so that the same amount of EB2-GFP and EB232-GFP bound the microtubule tip did not change the peak distance to EB3 (Fig. S6). Although reducing EB3 levels brought EB3 and EB2 distributions closer (Fig. S2A–C), this was due to the EB3 distribution shifting away from the tip (Fig. S2D,E), in line with published findings for EB1 (Maurer et al., 2014), rather than EB2 moving forward due to reduced competition. Nevertheless, we noted that the EB232 comet profile was not overlapping with EB3 as did the EB32 chimera that contains the entire EB3 CH domain (Fig. 8C–G). Our kymograph images revealed a potential explanation as EB232 retained the high affinity for GMPCPP-stabilised seeds that is typical for EB2, whereas EB32 did not (Fig. 8H). The N-terminal part of the CH domain contains only one cluster of three amino acids that are in proximity to tubulin and changed in EB2 (Fig. S5). Thus our results are consistent with the idea that V10I, N14T and L15M, which contact both α- and β-tubulin near the interdimer interface, are likely to mediate the increased GMPCPP microtubule binding of EB2 (Fig. S5).

Finally, to determine whether these structural determinants are of physiological relevance to the distinct EB binding in cells, we expressed EB2-GFP and EB2_IKFE_-GFP in a RPE1 EB3tdTomato cell line. EB2_IKFE_ carries point mutations (V182I, R143K, L147F, D148E) to revert four conservative amino acid changes present in the contact interface with the right-hand-side protofilament to those present in EB1 and EB3 because these were the key residues we implicated in the increased tip binding affinity and preference for pure GTPγS microtubules. Simultaneous two-colour imaging allowed analysing the position of the two EB2-GFP variants relative to EB3-tdTomato (Fig. 8I–L). Our data show that similarly to endogenous EB2 (Fig. 1), the EB2 peak position is shifted away from the microtubule tip by ∼280 nm relative to EB3 (Fig. 8I–J). The EB2 mutant showed a tip-tracking behaviour that is indistinguishable from that of EB3 (Fig. 8K–L). These findings support the idea that subtle charge-preserving amino acid changes fine-tune EB binding specificity, and we propose that tip tracking of EBs in spatially distinct zones is a result of different binding affinity profiles to the evolving nucleotide state environment at the microtubule tip, in conjunction with competition of the three EB paralogues for a subset of the available binding sites.

## DISCUSSION

We have shown that the mammalian EB paralogues EB1, EB2 and EB3 sense the nucleotide state of two adjoining tubulin subunits. Our study is in agreement with previous work that showed EB1 to bind preferentially to microtubule lattices containing GTP analogues (Zanic et al., 2009; Maurer et al., 2011), and with high-resolution cryo-electron microscopy data that show the EB3 CH domain to bind at the interface of four tubulin dimers in proximity to the exchangeable nucleotide binding site of two β-tubulins (Zhang et al., 2015). This explains how EBs can sense the nucleotide composition of the lattice with such sensitivity. In our mixed-lattice experiments, both EB1 and EB3 are highly sensitive to decreasing the GTPγS-tubulin content in the lattice in a way that suggests the simultaneous recognition of the nucleotide state of two or more tubulin subunits. Although a single CH domain can only conceivably sense the state of two subunits, we have to consider that EBs are dimeric proteins. It is currently unclear whether both CH domains form a composite binding site (Buey et al., 2011) or whether each CH domain can occupy a separate binding site. If we only consider the canonical binding sites at the interface of four tubulin dimers, then these two sites can either be in neighbouring protofilament grooves (as shown in Fig. S5) or along the same groove. In the former case, a row of three adjoining GTPγS-tubulins would be required to form the ideal binding site; in the latter case, two pairs – i.e. a quartet – are required. Our data show a distribution for EB3 that most closely matches the theoretical distribution of GTPγS-tubulin triplets in our mixed-lattice experiments (Fig. 7C), which favours the separate lateral binding site model. The only structural study looking at a dimeric EB protein binding to microtubules found Mal3 in a single row along the microtubule seam, and the authors suggested that CH domains bind to separate longitudinal binding sites (Sandblad et al., 2006). There is, however, a controversy as to whether EBs bind exclusively to the seam (Sandblad et al., 2006; des Georges et al., 2008) or are excluded from the seam (Maurer et al., 2012; Zhang et al., 2015; von Loeffelholz et al., 2017). The observed density of EBs at growing microtubule ends in cells (Seetapun et al., 2012) cannot be obtained by seam binding alone. Our data supporting a dual-nucleotide-recognition model are also not consistent with seam binding, as the binding site would be adjacent to one intradimer surface with nonexchangeable GTP bound to α-tubulin, in addition to one interdimer surface at which EBs could sense the nucleotide state of β-tubulin. The recent observation that the yeast EB Bim1 binds with a 4 nm repeat at the canonical binding sites near the interdimer interfaces, as well as near the intradimer interfaces (Howes et al., 2017), suggests that there might be an additional binding site accessible to some proteins of the EB family, providing a potential explanation for the different apparent saturation binding of different EB family members we observed (Fig. 4; Fig. S2). Future studies into the relationship of the two CH domains in microtubule binding and a high-resolution structure of EB dimers on the microtubule lattice will be required to understand how native dimeric EBs bind to the microtubule tip.

Our study raises the question of the structural determinants sensed by the EBs and leading to their different binding preferences. It is clear from previous work, and this study, that the EB binding site is nucleotide dependent. Recent cryo-electron microscopy structures from microtubules in different nucleotide states show that the most pronounced effect of GTP hydrolysis is a longitudinal compaction of the microtubule lattice by 1.5 Å per dimer (Alushin et al., 2014; Zhang et al., 2015, 2018; Manka and Moores, 2018). Yeast GTPγS microtubules are already partially compacted, but Bim1 causes further compaction and reduction of the dimer twist to both GTPγS microtubules and dynamic microtubules (Howes et al., 2017). In the presence of EB3, mammalian GMPCPP microtubules are compacted too, and EB3 mediates hydrolysis of GMPCPP (Zhang et al., 2015). EB3 also introduces a negative dimer twist into GTPγS-microtubules (Zhang et al., 2018). Together with the observations that EBs control the protofilament number, the length of taper at microtubule tips and mechanically stiffen the microtubule (Vitre et al., 2008; Lopez and Valentine, 2014; Zhang et al., 2015), this suggests that EBs not only sense, but also modify, the microtubule structure upon binding. This makes answering the question as to which structural properties determine the relative affinity of EB proteins to different nucleotide states a very challenging problem. However, it is interesting to note that the conformational changes triggered by EB1 and EB3 that result in a dose-dependent shift of their binding site closer to the microtubule tip (Fig. S2) (Maurer et al., 2014) seem not to be sensed by EB2.

The current thinking in the field is that GTPγS microtubules mimic the structure of a hydrolysis intermediate, such as GDP/P_i_-tubulin. This is based on the observation of a compacted lattice structure, the slow assembly of GTPγS-tubulin and being the favourite substrate for EB binding. The only caveat is that the recently reported structure for GTPγS-microtubules is more similar to GDP microtubules than GDP/P_i_ microtubules (Manka and Moores, 2018). It has also been argued that EBs detect the GTP-tubulin cap (Seetapun et al., 2012; Duellberg et al., 2016), but this is inconsistent with a number of observations in this study and in the existing literature: the EB comet is not right at the microtubule tip, which is apparent if compared with the tubulin signal (Fig. 3) (Maurer et al., 2014), as well as with XMAP-215, a bona fide marker for the microtubule tip (Nakamura et al., 2012; Maurer et al., 2014). In addition to GTPγS, EBs also bind preferentially to microtubules assembled with GDP/BeF_n_ (Maurer et al., 2012), a structural mimic for GDP/P_i_. The nucleotide state distribution for GDP/P_i_ in the microtubule lattice is predicted to be in the shape of an EB comet (Fig. 5) and involves GTP hydrolysis to form and phosphate release to decay. Our findings that EB1 and EB3 strongly prefer binding pure GTPγS lattices suggests that their favourite binding sites are those flanked by two GDP/P_i_-tubulins. This would still reconcile with the observation that EB comet size correlates with microtubule assembly speed and microtubule stability (Fig. 2) (Duellberg et al., 2016), as the prevalence of stabilising GTP-tubulin and of GDP/P_i_ pairs correlate. It needs to be noted, however, that EBs do not interact with the nucleotide itself, but rather sense the conformation of tubulin in different nucleotide states. This has been beautifully illustrated in a study using a yeast tubulin mutation that uncouples GTP hydrolysis from the associated conformational changes in tubulin (Geyer et al., 2015). Under those conditions, microtubules are highly dynamic and GTP hydrolysis occurs rapidly, but Bim1 decorates the microtubule lattice rather than being restricted to the growing microtubule tip (Geyer et al., 2015). Likewise, on *S. pombe* microtubules that do not show lattice compaction upon GTP hydrolysis, Mal3 has difficulties distinguishing the lattice and the tip of the microtubule (von Loeffelholz et al., 2017).

Our data show that EB2 prefers a different nucleotide composition of the microtubule lattice than EB1 and EB3, which is mediated through a number of conservative amino acid substitutions at the interaction surface with the right-hand-side protofilament. Introducing just four of the amino acids from EB3 in these positions renders the protein to an EB3-like tip tracker in cells. This has the following implications: binding to cargo proteins via the C-terminal tail of EBs seems not to drive the spatially distinct localisation as the EB2 mutant retains its linker and tail and thus EB2-specific cargo interactions. Also, truncated EB1 and EB3 constructs in which the C-terminal tail region was replaced with a leucine zipper have a microtubule-binding behaviour indistinguishable from the respective full-length proteins *in vitro* (Fig. 7) and in cells. Competitive binding is likely to contribute to spatially distinct binding in cells as EB2 binds closer to the tip when EB1 is reduced (Fig. 1). However, competition cannot explain everything: although EB2 competes with EB3 from the microtubule tip (Fig. 8A), adjusting EB2 protein levels 4-fold did not change its relative position to EB3 (Fig. S6). Moderate depletion of EB1 or EB3 does not result in a forward shift of EB2 in cells (data not shown). Furthermore, the same peak distance between 100 nM EB2 and EB3 was observed when it was measured for individual EBs relative to the microtubule tip or both proteins relative to each other when they were present simultaneously (Fig. 3). However, EB2 and EB3 positions changed relative to each other when EB3 was added at lower concentrations as the EB3 distribution shifts relative to the microtubule tip in a dose-dependent manner (Fig. S7). EB1 behaves the same, and this behaviour is explained by EB1 changing tubulin conformation to accelerate the formation and decay of its binding site (Maurer et al., 2014). Our data suggest that the EB2 position does not respond to that conformational change. In our mixed-lattice experiments, EB2 prefers lattices that contain GMPCPP and GTPγS. This cannot imply that EB2 prefers binding to GTP-tubulin and GDP/P_i_-tubulin pairs at the microtubule tip as these are invariably positioned more proximal to the tip than GDP/P_i_-tubulin pairs (Fig. 5), which are the most likely binding site for EB1 and EB3. Given the extended distribution of EB2 in cells and that we also observed increased EB2 binding to mixed GMPCPP/GDP lattices and comparable affinity for pure GTPγS and GTPγS/GDP pairs, it is more likely that EB2 is predominantly found at sites flanked by GDP/P_i_-tubulin and GDP-tubulin in cells. It is apparent from our experiments that all three mammalian EB paralogues bind to microtubule lattices with a range of nucleotide compositions and have distinct profiles of relative affinities for these. Our results are consistent with a model in which EB2 is the most competitive binder to sites further distal from the tip that only contain a low density of GTP or GDP/P_i_-tubulin, whereas EB1 and EB3 preferentially bind to a region of the microtubule with a high content of GDP/P_i_. However, all three EBs have an identical microtubule-binding interface with the left-hand-side protofilament and bind efficiently to GTPγS-microtubule lattices. Therefore, competition for sites at growing microtubule tips occurs and total levels of EBs bound at the microtubule tip are reduced in the presence of competing EBs both in cells and *in vitro* (Figs 1 and 8) (Straube and Merdes, 2007). In addition, EBs modulate the microtubule structure and future studies will be required to understand how the different EB paralogues affect microtubule structure differentially as this might affect each other's binding beyond competition for the same site.

A caveat of our *in vitro* experiments is that we cannot reproduce the magnitude of the spatial shift between EB3 and EB2, nor the small lead of EB1 over EB3 that we observed in cells. In these experiments, the microtubule assembly rate is significantly lower than in cells and one would expect that the nucleotide distributions that we show in Fig. 5 become increasingly spaced apart the faster microtubules assemble. Thus, different nucleotide-dependent binding sites should also be further apart when microtubules grow faster. However, if we modulate assembly speed over a 3-fold range by varying the free tubulin concentration, we do not see an increase in the peak distance of EB3-mCherry and EB2-GFP (data not shown). There is also no correlation between peak distance and assembly speed measured for the individual growth phases in any of our dual-colour *in vitro* experiments (data not shown). There are two possible explanations for this: either the EB2 shift is not nucleotide dependent or the formation of the EB2 binding site is accelerated by EB3 at the same rate as microtubule assembly. The latter seems feasible given that faster-assembling microtubules recruit more EB3 (Fig. 2), and that we could recapitulate the concentration-dependent acceleration of the maturation steps forming and deconstructing the EB1 binding site (Maurer et al., 2014) for EB3 in this study (Fig. S7). This acceleration might not occur to the same extent in cells owing to the presence of other cellular factors. We can also only speculate what drives the separation of EB1 and EB3 in cells and why EB2 only moves into the sites freed by EB1 upon EB1/3 co-depletion. One possibility is offered by the observation that EB1 seems not to be sensitive to the longitudinal curvature of microtubules and was found to decorate both outward curved and straight sheets as well as closed lattice regions to a comparable extent (Bechstedt et al., 2014; Guesdon et al., 2016). Whether EB2 or EB3 are sensitive to longitudinal curvature features remains to be tested. If EB3 was to prefer straight microtubules, it would provide a possible explanation for the avoidance of the zone closer to the tip. As we controlled for taper in our single EB experiments by including only blunt microtubules into the analysis, any taper-related changes in EB positioning might not have revealed themselves. We consider this unlikely, because dual-colour experiments with EB1 and EB3 did not show a proximal shift of EB1 either. Thus, additional cues that were not reproduced in our reconstitution experiments such as post-translational modifications of EBs in cells, other MAPs decorating different regions of the tip or controlling the lattice structure might modulate EB binding in cells.

Our study does demonstrate that the higher microtubule-binding affinity of EB3 observed in cells by us (Fig. S2) and others (Stepanova et al., 2003) is an intrinsic property (Figs 2 and 3). Thus, cells have at their disposal three EBs with different microtubule-binding properties. We already know that in several cell types, EB2 and EB3 expression is regulated during differentiation. Many polarised cell types, such as neurons and muscle, upregulate EB3 upon differentiation, whereas EB2 is downregulated upon myoblast and apicobasal epithelial differentiation (Nakagawa et al., 2000; Straube and Merdes, 2007; Goldspink et al., 2013). Thus, cells seem to use transcriptional control to express different combinations of EB proteins and thereby control the composition of the plus-tip network. Given the different properties we describe here, cells will be able to control the extent of the EB zone and position EB interactors in spatially distinct areas on the microtubule tip. This could affect how EB interactors regulate microtubule dynamics; for example, a position further away from the tip might allow a rescue factor to re-establish microtubule growth shortly after a catastrophe occurred. Likewise, tip tracking of proteins that destabilise the microtubule tip might be differently effective if bound to an EB at a different distance from the tip. It has been shown *in vitro* that EB3 can promote tip tracking of the depolymerising kinesin MCAK and at the same time protect the microtubule to some extent from depolymerisation by its cargo (Montenegro Gouveia et al., 2010). An interesting question for the future would be whether tip tracking on a different EB would change the activity of MCAK. The spatially separate positioning might also result in zones facilitating different interactions and signalling events within the tip-tracking network. An example of an EB-facilitated interaction is that of Navigator and the Rho-GEF Trio, which is important for Rac1-driven neurite outgrowth (van Haren et al., 2014). Thus our study opens new questions into the spatial organisation of signalling events that are regulated by the +tip network.

## MATERIALS AND METHODS

### Cell culture and immunostaining

Human retinal pigment epithelial (RPE1) cells immortalised with hTERT (Clontech) were grown in Dulbecco's modified Eagle medium (DMEM)/F-12 medium (D6421, Sigma-Aldrich) containing 10% fetal bovine serum (FBS), 2.3 g/l sodium bicarbonate, 2 mM L-glutamine, 100 U/ml penicillin and 100 µg/ml streptomycin at 37°C, 5% CO_2_ in a humidified incubator. The RPE1 ET28 cell line (Theisen et al., 2012) stably expressing EB3-tdTomato was grown in RPE medium supplemented with 500 µg/ml Geneticin. Murine myoblasts (C2C12) were grown in DMEM GlutaMAX medium (Invitrogen) containing 10% FBS, 100 U/ml penicillin and 100 µg/ml streptomycin in rat tail collagen (C3867, Sigma-Aldrich)-coated dishes at 37°C, 5% CO_2_ in a humidified incubator. Cells were checked for mycoplasma infection monthly using MycoSensor PCR Assay Kit (Agilent Genomics). For immunofluorescence staining, RPE1 cells were seeded onto coverslips coated with 10 µg/ml fibronectin (F1141, Sigma-Aldrich) and C2C12 cells were seeded onto collagen-coated coverslips. After 24 h, cells were fixed in −20°C pre-cooled methanol and stained with 1:100 mouse anti-EB1 (BD Biosciences, cat. 610534, lot 33974), 1:1000 rat anti-EB1 (KT51, Absea Biotechnology, cat. 010811B11, lot 09123114916), 1:400 rat anti-EB2 (KT52, Absea Biotechnology, cat. 010614A11, lot 05020536605) and 1:500 rabbit anti-EB3 (Komarova et al., 2005) antibodies. Secondary antibodies were cross-absorbed donkey anti-mouse, anti-rat and anti-rabbit antibodies conjugated to Alexa Fluor 488, Alexa Fluor 594 or Alexa Fluor 647 (A-21202, A-21203, A-31571, A-21205, A-21206, A-31573, A-21209; Invitrogen). For each dataset, all three EB proteins were stained simultaneously using different combinations of secondary antibodies. Image stacks were acquired on a Perkin Elmer Ultraview spinning disk confocal microscope using a 100×/1.4 NA objective, 405 nm, 488 nm, 561 nm and 640 nm lasers and an Orca-R2 camera (Hamamatsu) under the control of Volocity software (Perkin Elmer). For live-cell imaging, 6000 cells were seeded onto a glass-bottom dish coated with 10 µg/ml fibronectin, transfected with Fugene6 and imaged simultaneously in GFP and RFP channels using 488 nm and 561 nm excitation lasers and two Orca-R2 cameras. Images were corrected for chromatic aberration using images from 200 nm TetraSpeck^TM^ beads acquired on the same day using the ImageJ plugin ‘Descriptor-based series registration’ (Preibisch et al., 2010).

For three-colour data, line scans from microtubule ends were obtained using the Plot profile function of ImageJ in all three EB channels, aligned at the pixel closest to the midpoint between the first half-maximal points of the EB1 and EB3 signals and averaged. To remove any effects caused by different fluorophores, mean distributions from experiments using different fluorophore-EB combinations were averaged for the each of the two different cell lines. For live-cell data, line scans were obtained from comets that were actively growing and not touching the cell cortex; intensity data were aligned at the first half-maximal point in the EB3 signal and averaged using a custom MATLAB script available at http://mechanochemistry.org/Straube/LineScans.m.

### Cloning and protein purification

EB1 (NM_007896), EB2 (NM_153058) and EB3 (NM_133350) open reading frames (ORFs) were amplified from random primed cDNA from C2C12 cells (Straube and Merdes, 2007), introducing *Nde*I and *Eco*RI restriction sites. GFP was amplified from pEGFP-C1 to introduce *Eco*RI and *Not*I restriction sites. EB and GFP fragments were ligated to pET22b opened with *Nde*I and *Not*I. A resulting frameshift was corrected by opening with *Eco*RI, mung bean nuclease treatment and re-ligation of the vector. This allowed expression of EB-GFP-6xHis constructs. GFP was replaced by mCherry to obtain EB1/3-mCherry-6His. EB1CH-LZ was described previously (Grimaldi et al., 2014). The respective EB3 construct was obtained by introducing the *Mlu*I site following P137 in EB3 (which is the corresponding position to EB1 P139) and fusion to the leucine zipper from yeast GCN4 as *Mlu*I-*Bam*HI fragment upstream of GFP and 6xHis. The EB2-EB3-EB2 chimera was cloned using conserved restriction sites, namely *Pfl*MI centred at EB3 F47 and EB2 F89 and *Bg*lII at EB3 I145/EB2 I187 to swap domains. For mammalian expression, EB ORFs were amplified by PCR from cDNA and cloned as *Sac*I to *Sac*II fragments into pEGFP-N1. The EB2_IKFE_ mutant was generated from the EB2-GFP plasmid by PCR-based mutagenesis using the following reverse-priming oligonucleotides (nucleotides different from EB2 ORF are highlighted in bold italic): 5′-CACTGGGAT***G***A***T***CTTATCAACATTC-3′ and 5′-CACTGAATAAA***C***TC***G***A***A***GTTGTCTTGGAA***CTT***CCCTTTCAC-3′. The mutated EB2 was exchanged using *Pfl*MI and *Sac*II restriction enzymes. All plasmid sequences were verified by DNA sequencing. Constructs were expressed in *Escherichia* *coli* BL21 (DE3) at 18°C. Bacteria were lysed in binding buffer (50 mM KPO_4_ buffer pH 7.2, 400 mM NaCl, 2 mM MgCl_2_, 2 mM 2-Mercaptoethanol, 12 mM imidazole) supplemented with 0.1% Triton X-100, 1 mg/ml lysozyme and 1 mM phenylmethylsulfonyl fluoride by sonication. The high-speed supernatant was incubated with Ni-NTA agarose, washed with binding buffer containing 20 mM imidazole and eluted with 250 mM imidazole. The EB-containing fractions were loaded onto a Superdex200 16/60 column (GE Healthcare) and eluted using binding buffer without imidazole. The peak fractions were combined, concentrated using vivaspin columns (Sartorius), supplemented with 20% glycerol, snap frozen and stored in liquid nitrogen. Protein concentration was determined by measuring absorption at 280 nm as well as quantification of Coomassie Blue staining and Sypro Red fluorescence of bands in polyacrylamide gels.

### *In vitro* microtubule-binding assays

Tubulin was prepared from pig brains according to published protocols (Gell et al., 2011). Note that we freeze purified tubulin without prior addition of glycerol, as we observed increased EB binding to microtubules in the presence of >1.5% glycerol. Labelled tubulin was from Cytoskeleton Inc., nucleotides were from Jena Biosciences and all other chemicals were from Sigma-Aldrich, unless indicated. Microtubule seeds were assembled from tubulin, biotin-tubulin and HiLyte647-tubulin at a molar ratio of 25:1:2 in the presence of 1 mM GMPCPP in MRB80 (80 mM PIPES, pH 6.8 with KOH, 1 mM EGTA, 4 mM MgCl_2_) for 1 h at 37°C, diluted 20-fold with MRB80+2 µM Taxol and stored at room temperature. For the binding assays, GTPγS extensions were made onto GMPCPP seeds using an elongation mix containing 12 µM tubulin, 1 µM X-Rhodamine tubulin, 0.5 µM biotin-tubulin and 1 mM GTPγS in MRB80, and incubated for 1 h at 37°C. A 100 µm deep-flow chamber was made from a slide and a hydrochloric acid-treated coverslip using double-sided tape (Scotch 3M) and passivated with PLL-PEG-50% biotin (Susos AG). Seeds were attached to this surface using streptavidin, then any Taxol and unattached seeds were washed out before blocking with 1 mg/ml κ-casein. A reaction mix containing 12 µM tubulin, 50 mM KCl, 1 mM GTP, 0.6 mg/ml κ-casein, 0.2% methyl cellulose, 4 mM DTT, 0.2 mg/ml catalase, 0.4 mg/ml glucose oxidase and 50 mM glucose in MRB80, supplemented with EB proteins or buffer, was clarified for 8 min at 190,000 ***g*** in an airfuge (Beckman Coulter). The supernatant was then added to the flow chamber, which was sealed with candle wax. Microtubule assembly and EB binding were observed on an Olympus TIRF system using a 100×/1.49 NA objective, 1.6× additional magnification, 488 nm, 561 nm and 640 nm laser lines, and a Hamamatsu ImagEM-1K back-illuminated EM-CCD camera under the control of xCellence software. Resulting spatial resolution of images was 81 nm/pixel. Microtubule binding was measured using ImageJ: GMPCPP, GTPγS and GDP microtubules were traced by line segments and the average intensity in a 3-pixel-wide box along this line was determined; tip intensity was measured using a 3×3 pixel box at the time point the tip was brightest during a 100 s movie. To analyse EB localisation relative to the microtubule tip, 17% HiLyte488-tubulin was included in the protein mix to label microtubules uniformly. Images of microtubules and EBs were collected sequentially at 500 ms intervals and analysed entirely using an algorithm developed in MATLAB (as described below). For fluorescence recovery after photobleaching experiments, diffraction-limited spots of a 405 nm laser were exposed for 20 ms on EB comets during continuous imaging with 561 nm in TIR mode at 200 ms or 300 ms cycle time using ‘fire on click’ mode. Only comets that grew at the same speed during the recovery period as before the bleach event were included in the analysis. Data were normalised to 1 at the last pre-bleach image and 0 at the first postbleach image before averaging. An exponential curve was fitted to the postbleach intensity values using Origin Pro 8.51 (Originlab). For mixed-lattice experiments, tubulin was equilibrated on ice for 1 h in the presence of 5% labelled tubulin (either with HiLyte647 or X-rhodamine) and 1 mM nucleotide (either GTP, GMPCPP or GTPγS) to allow for complete exchange of the nucleotide in the E-site. Nucleotide-equilibrated tubulin was mixed at 1:5 or 1:1 ratios or left pure and immediately placed at 37°C for 1 h to allow assembly. Free nucleotide and tubulin were removed by centrifugation and re-suspension of microtubules in MRB80+2 µM Taxol. Mixed-lattice microtubules were used within 6 h from assembly for binding experiments. Images were acquired within 15 min from adding EBs to the chamber in the absence of free nucleotide to prevent EB-stimulated nucleotide hydrolysis or artefacts from nucleotide binding to EBs (Gireesh et al., 2014, Zhang et al., 2015). Binding to mixed lattices was conducted in MRB80 plus 50 mM KCl, 0.6 mg/ml κ-casein and an oxygen scavenger system (4 mM DTT, 0.2 mg/ml catalase, 0.4 mg/ml glucose oxidase and 50 mM glucose) for all proteins except EB1, which was assayed at a reduced salt concentration of 5 mM KCl.

### Comet shape and position analysis

Comet shape analysis was performed on kymographs that were generated using the ImageJ plugin by Arne Seitz (http://biop.epfl.ch/TOOL_KYMOGRAPH.html). Kymographs were manually cropped to segments of linear growth in ImageJ and analysed using custom MATLAB scripts, which are available on our laboratory website (http://mechanochemistry.org/Straube/DualColourKymograph_Analysis.zip) and the CMCB git hub (https://github.com/cmcb-warwick). The EB comet was detected by least-square-regression linear fit through the locations of the first 90% maximal intensity values for each time point. To exclude erroneous high intensity spots in the image field, we used the fitted line to create a ±5 pixel confidence interval. We repeated the above step restricting the location of the tip to the confidence interval. The resulting line of best fit was rejected if the residual error was greater than 1 pixel (81 nm). If accepted, this line was used as the reference to align EB comet data in time. To allow more precise alignment, spatial resolution was increased 10-fold to 8.1 nm/pixel by cubic interpolation. The intensity profiles were aligned at the position on the reference line rounded to the nearest pixel for each time point and averaged over the linear growth period. We then subtracted the average background before the microtubule tip and normalised the curve by dividing by the maximum. Curves from different microtubules were superaveraged using the first half-maximal point. Data were pooled from five experiments performed at a range of concentrations (25–400 nM for EB3, 50–600 nM for EB2 and 100–800 nM for EB1) within which comet shape was not significantly altered. The total area under the comet was calculated in two parts: (1) until the 85% maximum value following the peak, area was calculated directly from the curve values; (2) from the second 85% point, an exponential curve was fitted to the comet decay and area determined as the area under the fitted curve.

To determine the peak position of two EB proteins relative to each other, timelapse images of EB3-mCherry and a second EB protein as a GFP fusion were collected at 1 fps, sequentially, exactly 500 ms apart. Kymographs were cropped to linear growth phases, interpolated 2-fold in time, and the first line of the first channel kymograph and the last line of the second channel kymograph were then removed to correct for time offset due to sequential imaging. Data for both channels were analysed as for the comet shape data above, with all manipulations calculated for the EB3-mCherry channel and applied to the GFP channel. Data from different growth phases were superaveraged and peak positions determined as the maximal intensity of the averaged curves. In addition, peak distances were determined from the average intensity profiles for each growth phase. Data in the main figures were pooled from three independent experiments.

To determine localisation of the EB comet relative to the microtubule end, we first precisely determined the localisation of the microtubule and its plus end in the image stacks. Microtubules suitable for analysis (i.e. sufficiently isolated from other microtubules that could interfere with the analysis) were selected manually in the first frame of the image stack. Based on this selection, a substack was cropped in both the microtubule and EB channel and saved for further analysis. The image was transformed using reflection and transposition to orient each microtubule with the seed end closest to the origin and the microtubule angle between 0 and 45°. The microtubule backbone was identified by fitting a Gaussian to the intensity profile of each column in the microtubule image and fitting either a straight line, or in the case of poor fit, a cubic curve through the peak positions of these Gaussians. Using the microtubule backbone as a reference, a new image (21 pixels high) was created by bi-cubic interpolation for each time point. The new image has the microtubule running through the vertical centre of the image. To extract microtubule intensity, the intensity of the central 9 pixels was averaged and background corrected by subtraction of the mean intensity of the 8 extreme pixels (4 on either side). The microtubule end position *µ* and the variance *σ* were determined by fitting a Gauss error function (Fig. 3B,C). From the end positions, microtubule length was calculated for all time points and phases of microtubule growth identified by an iterative segment line fit algorithm. To do this, a least squares fitted line was recursively divided to include the point of greatest distance from the line until the average perpendicular distance was 20 nm or less. Phases of at least 10 s length and an average growth velocity *v_growth_* between 10 nm/s and 30 nm/s were kept for analysis. This ensured that only microtubules in a stable growth state were compared, and alleviated differences in microtubule growth stimulation by the different EBs. Within these growth phases, we only considered time points for which the Gauss error function could be fit with a variance *σ* between 50 nm and 200 nm, as this guaranteed a precise determination of the end position and excluded microtubules with a long taper. For these time points, EB intensity data along the microtubule backbone were extracted in the same way as for the microtubule intensity from a 21-pixel-high image. Using *µ* for the microtubule channel and *µ-0.5•v_growth_* for the EB channel as a reference, intensity values were interpolated in 8 nm intervals. This aligned all microtubule ends and corrected for temporal shift between images. Data were pooled from three independent experiments.

To benchmark the accuracy of our algorithm, we generated synthetic images of microtubules using a previously published strategy (Demchouk et al., 2011), with the modifications that we generated images of continuously growing microtubules, downsampled data to the pixel size of our imaging system (81 nm), used a Poisson distribution to sample intensity values based on the 1.5 Hilyte488 dye to tubulin dimer ratio as in our experiments, convolved the synthetic images with a Gaussian with a standard deviation of 130 nm (representing the point spread function determined experimentally from our imaging system) and added real imaging noise that we acquired from microtubule-free areas in our experimental chambers to achieve realistic signal-to-noise ratios (SNRs). Each synthetic microtubule was simulated as an image stack with microtubule length varying from 2 µm to 4 µm and back at 50 nm per frame; intensity values were comparable to experiments with an SNR of 6 and variable fractions of labelled tubulin of 6%, 12%, 18%, 25%, 35% and 50%. In a separate dataset, microtubules were simulated with 18% labelled tubulin at varying SNRs of 1, 3, 6, 9, 12 and 15. Gauss error functions were fitted to both ends of the microtubule and the length of the microtubule was determined. This value was compared with the simulated microtubule length. For each condition, 10 synthetic image stacks with 81 frames were generated and analysed. To determine the SNR of our images, we measured the average intensity of the microtubule backbone, subtracted the average intensity image background and divided the result by the standard deviation of the image background.

### Calculation of paired nucleotide distributions

To determine the distribution of the pairwise combinations of GTP, GDP/Pi and GDP we first calculated the distribution of GTP, GDP/Pi and GDP as a function of the distance from the microtubule tip. We assume a 13-protofilament blunt-ended microtubule and uncoupled first-order kinetics for both GTP hydrolysis and phosphate release. This is described with the following equations: *dT/dL=-k_1_•T*, *dP/dL=k_1_•T-k_2_•P* and *dD/dL=k_2_•P*, with *T*, *P* and *D* being the number of tubulin subunits in a layer containing a GTP, GDP/Pi or GDP, respectively. *L* is the number of subunits from the tip; *k_1_* and *k_2_* are reaction constants for GTP hydrolysis and phosphate release, respectively. These functions were solved numerically using an explicit Runge–Kutta (4,5) formula (Dormand and Prince, 1980). The distributions of pairwise combinations of nucleotides were calculated analytically by converting the numerical results from the Runge–Kutta method into probabilities and calculating the probability of each of the six pairwise combinations: *TT*, *TP=PT*, *PP*, *PD=DP*, *TD=DT* and *DD*. These were then multiplied by 13 to give a representative number of lateral dimer-dimer interfaces per tubulin layer. To obtain a representative image of how these curves would look in an experiment, the point-spread function was obtained experimentally by fitting Gaussians to cross-sections of Hilyte488-labelled microtubules on our TIRF setup. Dimer distribution curves were then convolved by multiplication with a Gaussian with a standard deviation of 130 nm and normalised to maximal intensity. The MATLAB script to simulate paired nucleotide distributions is available at http://mechanochemistry.org/Straube/PairedNucleotides.m.

### HPLC analysis of nucleotide composition in microtubule lattice

Microtubules were assembled as for mixed-lattice experiments, pelleted through a 30% sucrose cushion in 20 mM KPO_4_ buffer pH7, resuspended in 20 mM KPO_4_ pH7 and placed on ice. Nucleotides were extracted as previously described (Dye and Williams, 1996) by adding ice-cold perchloric acid to a final concentration of 5%, vortexing for 5 s and 10 min, and incubating on ice. Precipitated protein was removed by 10 min centrifugation at 20,000 ***g*** at 4°C. The samples were neutralised using 1 M KH_2_PO_4_ and 3 M KOH, incubated on ice for 10 min and then precipitates were removed by centrifugation as before. Cleared supernatants were analysed by isocratic ion-pairing reverse-phase chromatography on octadecylsilica (ACE C18 5 μm, 250×4.6 mm), with detection at 254 nm, using 150 mM KH_2_PO_4_/KOH pH5.9 supplemented with 1.5 mM tetrabutylammonium bromide as running buffer (Perrone and Brown, 1984). The injection volumes were 50 μl and the flowrate was 1 ml/min. Peaks were identified by comparison of their retention times to nucleotide standards processed in parallel to the microtubule samples. Chromatography profiles were subtracted with a baseline measured at 300 nm and plotted using MATLAB. Peak areas were analysed using ChromNAV software (Jasco UK) and relative nucleotide content was determined as a ratio of peak areas from mixed and pure microtubules after normalisation to either the total area of the nucleotide peaks or the GTP peak as two alternative means to control for unequal amount of microtubule assembly in the different conditions.

### Statistical analysis

Statistical hypothesis testing (one-sample Student's *t*-test, two-sample Student's *t*-test, paired Student's *t*-test, two-sample Kolmogorov–Smirnov test and Mann–Whitney *U*-test, as appropriate) and curve fitting was performed using Origin Pro 8.5 (Originlabs), MATLAB (MathWorks) or R (https://www.R-project.org/). Means were considered to be statistically significantly different when *P*<0.05. Error bars in graphs show standard deviation (s.d.) or standard error of the mean (s.e.m.) as indicated. Number of experiments and measurements are indicated in the Materials and Methods and/or figure legends.

## Supplementary Material

Supplementary information

## References

[JCS219550C1] Alushin G. M., Lander G. C., Kellogg E. H., Zhang R., Baker D. and Nogales E. (2014). High-resolution microtubule structures reveal the structural transitions in alphabeta-tubulin upon GTP hydrolysis. *Cell* 157, 1117-1129. 10.1016/j.cell.2014.03.053 24855948 PMC4054694

[JCS219550C2] Bechstedt S., Lu K. and Brouhard G. J. (2014). Doublecortin recognizes the longitudinal curvature of the microtubule end and lattice. *Curr. Biol.* 24, 2366-2375. 10.1016/j.cub.2014.08.039 25283777

[JCS219550C3] Beinhauer J. D., Hagan I. M., Hegemann J. H. and Fleig U. (1997). Mal3, the fission yeast homologue of the human APC-interacting protein EB-1 is required for microtubule integrity and the maintenance of cell form. *J. Cell Biol.* 139, 717-728. 10.1083/jcb.139.3.717 9348288 PMC2141698

[JCS219550C4] Bieling P., Laan L., Schek H., Munteanu E. L., Sandblad L., Dogterom M., Brunner D. and Surrey T. (2007). Reconstitution of a microtubule plus-end tracking system in vitro. *Nature* 450, 1100-1105. 10.1038/nature06386 18059460

[JCS219550C5] Bieling P., Kandels-Lewis S., Telley I. A., Van Dijk J., Janke C. and Surrey T. (2008). CLIP-170 tracks growing microtubule ends by dynamically recognizing composite EB1/tubulin-binding sites. *J. Cell Biol.* 183, 1223-1233. 10.1083/jcb.200809190 19103809 PMC2606963

[JCS219550C6] Bu W. and Su L.-K. (2003). Characterization of functional domains of human EB1 family proteins. *J. Biol. Chem.* 278, 49721-49731. 10.1074/jbc.M306194200 14514668

[JCS219550C7] Buey R. M., Mohan R., Leslie K., Walzthoeni T., Missimer J. H., Menzel A., Bjelic S., Bargsten K., Grigoriev I., Smal I. et al. (2011). Insights into EB1 structure and the role of its C-terminal domain for discriminating microtubule tips from the lattice. *Mol. Biol. Cell* 22, 2912-2923. 10.1091/mbc.e11-01-0017 21737692 PMC3154886

[JCS219550C8] Demchouk A. O., Gardner M. K. and Odde D. J. (2011). Microtubule tip tracking and tip structures at the nanometer scale using digital fluorescence microscopy. *Cell Mol. Bioeng.* 4, 192-204. 10.1007/s12195-010-0155-6 23002398 PMC3445660

[JCS219550C9] Des Georges A., Katsuki M., Drummond D. R., Osei M., Cross R. A. and Amos L. A. (2008). Mal3, the Schizosaccharomyces pombe homolog of EB1, changes the microtubule lattice. *Nat. Struct. Mol. Biol.* 15, 1102-1108. 10.1038/nsmb.1482 18794845 PMC2575238

[JCS219550C10] Dixit R., Barnett B., Lazarus J. E., Tokito M., Goldman Y. E. and Holzbaur E. L. F. (2009). Microtubule plus-end tracking by CLIP-170 requires EB1. *Proc. Natl. Acad. Sci. USA* 106, 492-497. 10.1073/pnas.0807614106 19126680 PMC2626730

[JCS219550C11] Dormand J. R. and Prince P. J. (1980). A family of embedded Runge-Kutta formulae. *J. Comput. Appl. Math.* 6, 19-26. 10.1016/0771-050X(80)90013-3

[JCS219550C12] Duellberg C., Trokter M., Jha R., Sen I., Steinmetz M. O. and Surrey T. (2014). Reconstitution of a hierarchical +TIP interaction network controlling microtubule end tracking of dynein. *Nat. Cell Biol.* 16, 804-811. 10.1038/ncb2999 24997520

[JCS219550C13] Duellberg C., Cade N. I., Holmes D. and Surrey T. (2016). The size of the EB cap determines instantaneous microtubule stability. *Elife* 5, e13470 10.7554/eLife.13470 27050486 PMC4829430

[JCS219550C14] Dye R. B. and Williams R. C. (1996). Assembly of microtubules from tubulin bearing the Nonhydrolyzable Guanosine triphosphate analogue GMPPCP [Guanylyl 5′-(β,γ-Methylenediphosphonate)]: variability of growth rates and the hydrolysis of GTP†. *Biochemistry* 35, 14331-14339. 10.1021/bi961070e 8916920

[JCS219550C15] Ferreira J. G., Pereira A. J., Akhmanova A. and Maiato H. (2013). Aurora B spatially regulates EB3 phosphorylation to coordinate daughter cell adhesion with cytokinesis. *J. Cell Biol.* 201, 709-724. 10.1083/jcb.201301131 23712260 PMC3664705

[JCS219550C16] Gell C., Friel C. T., Borgonovo B., Drechsel D. N., Hyman A. A. and Howard J. (2011). Purification of tubulin from porcine brain. *Methods Mol. Biol.* 777, 15-28. 10.1007/978-1-61779-252-6_2 21773918

[JCS219550C17] Geyer E. A., Burns A., Lalonde B. A., Ye X., Piedra F. A., Huffaker T. C. and Rice L. M. (2015). A mutation uncouples the tubulin conformational and GTPase cycles, revealing allosteric control of microtubule dynamics. *Elife* 4, e10113 10.7554/eLife.10113 26439009 PMC4728127

[JCS219550C18] Gireesh K. K., Sreeja J. S., Chakraborti S., Singh P., Thomas G. E., Gupta H. and Manna T. (2014). Microtubule +TIP protein EB1 binds to GTP and undergoes dissociation from dimer to monomer on binding GTP. *Biochemistry* 53, 5551-5557. 10.1021/bi5007942 25111064

[JCS219550C19] Goldspink D. A., Gadsby J. R., Bellett G., Keynton J., Tyrrell B. J., Lund E. K., Powell P. P., Thomas P. and Mogensen M. M. (2013). The microtubule end-binding protein EB2 is a central regulator of microtubule reorganisation in apico-basal epithelial differentiation. *J. Cell Sci.* 126, 4000-4014. 10.1242/jcs.129759 23813963

[JCS219550C20] Grimaldi A. D., Maki T., Fitton B. P., Roth D., Yampolsky D., Davidson M. W., Svitkina T., Straube A., Hayashi I. and Kaverina I. (2014). CLASPs are required for proper microtubule localization of end-binding proteins. *Dev. Cell* 30, 343-352. 10.1016/j.devcel.2014.06.026 25117684 PMC4133696

[JCS219550C21] Guesdon A., Bazile F., Buey R. M., Mohan R., Monier S., García R. R., Angevin M., Heichette C., Wieneke R., Tampé R. et al. (2016). EB1 interacts with outwardly curved and straight regions of the microtubule lattice. *Nat. Cell Biol.* 18, 1102-1108. 10.1038/ncb3412 27617931

[JCS219550C22] Hayashi I. and Ikura M. (2003). Crystal structure of the amino-terminal microtubule-binding domain of end-binding protein 1 (EB1). *J. Biol. Chem.* 278, 36430-36434. 10.1074/jbc.M305773200 12857735

[JCS219550C23] Honnappa S., John C. M., Kostrewa D., Winkler F. K. and Steinmetz M. O. (2005). Structural insights into the EB1-APC interaction. *EMBO J.* 24, 261-269. 10.1038/sj.emboj.7600529 15616574 PMC545816

[JCS219550C24] Honnappa S., Gouveia S. M., Weisbrich A., Damberger F. F., Bhavesh N. S., Jawhari H., Grigoriev I., Van Rijssel F. J. A., Buey R. M., Lawera A. et al. (2009). An EB1-binding motif acts as a microtubule tip localization signal. *Cell* 138, 366-376. 10.1016/j.cell.2009.04.065 19632184

[JCS219550C25] Howard J. and Hyman A. A. (2009). Growth, fluctuation and switching at microtubule plus ends. *Nat. Rev. Mol. Cell Biol.* 10, 569-574. 10.1038/nrm2713 19513082

[JCS219550C26] Howes S. C., Geyer E. A., Lafrance B., Zhang R., Kellogg E. H., Westermann S., Rice L. M. and Nogales E. (2017). Structural differences between yeast and mammalian microtubules revealed by cryo-EM. *J. Cell Biol.* 216, 2669-2677. 28652389 10.1083/jcb.201612195 PMC5584162

[JCS219550C27] Hyman A. A., Salser S., Drechsel D. N., Unwin N. and Mitchison T. J. (1992). Role of GTP hydrolysis in microtubule dynamics: information from a slowly hydrolyzable analogue, GMPCPP. *Mol. Biol. Cell* 3, 1155-1167. 10.1091/mbc.3.10.1155 1421572 PMC275679

[JCS219550C28] Isrie M., Breuss M., Tian G., Hansen A. H., Cristofoli F., Morandell J., Kupchinsky Z. A., Sifrim A., Rodriguez-Rodriguez C. M., Dapena E. P. et al. (2015). Mutations in either TUBB or MAPRE2 cause circumferential skin creases kunze type. *Am. J. Hum. Genet.* 97, 790-800. 10.1016/j.ajhg.2015.10.014 26637975 PMC4678434

[JCS219550C29] Jaworski J., Kapitein L. C., Gouveia S. M., Dortland B. R., Wulf P. S., Grigoriev I., Camera P., Spangler S. A., Di Stefano P., Demmers J. et al. (2009). Dynamic microtubules regulate dendritic spine morphology and synaptic plasticity. *Neuron* 61, 85-100. 10.1016/j.neuron.2008.11.013 19146815

[JCS219550C30] Jiang K., Toedt G., Montenegro Gouveia S., Davey N. E., Hua S., Van Der Vaart B., Grigoriev I., Larsen J., Pedersen L. B., Bezstarosti K. et al. (2012). A Proteome-wide screen for mammalian SxIP motif-containing microtubule plus-end tracking proteins. *Curr. Biol.* 22, 1800-1807. 10.1016/j.cub.2012.07.047 22885064

[JCS219550C31] Kirsch M. and Yarbrough L. R. (1981). Assembly of tubulin with nucleotide analogs. *J. Biol. Chem.* 256, 106-111. 7451427

[JCS219550C32] Komarova Y., Lansbergen G., Galjart N., Grosveld F., Borisy G. G. and Akhmanova A. (2005). EB1 and EB3 control CLIP dissociation from the ends of growing microtubules. *Mol. Biol. Cell* 16, 5334-5345. 10.1091/mbc.e05-07-0614 16148041 PMC1266430

[JCS219550C33] Komarova Y., De Groot C. O., Grigoriev I., Gouveia S. M., Munteanu E. L., Schober J. M., Honnappa S., Buey R. M., Hoogenraad C. C., Dogterom M. et al. (2009). Mammalian end binding proteins control persistent microtubule growth. *J. Cell Biol.* 184, 691-706. 10.1083/jcb.200807179 19255245 PMC2686402

[JCS219550C34] Lopez B. J. and Valentine M. T. (2014). Mechanical effects of EB1 on microtubules depend on GTP hydrolysis state and presence of paclitaxel. *Cytoskeleton (Hoboken)* 71, 530-541. 10.1002/cm.21190 25160006

[JCS219550C35] Manka S. W. and Moores C. A. (2018). The role of tubulin-tubulin lattice contacts in the mechanism of microtubule dynamic instability. *Nat. Struct. Mol. Biol.* 25, 607-615. 10.1038/s41594-018-0087-8 29967541 PMC6201834

[JCS219550C36] Maurer S. P., Bieling P., Cope J., Hoenger A. and Surrey T. (2011). GTPgammaS microtubules mimic the growing microtubule end structure recognized by end-binding proteins (EBs). *Proc. Natl. Acad. Sci. USA* 108, 3988-3993. 10.1073/pnas.1014758108 21368119 PMC3053978

[JCS219550C37] Maurer S. P., Fourniol F. J., Bohner G., Moores C. A. and Surrey T. (2012). EBs recognize a nucleotide-dependent structural cap at growing microtubule ends. *Cell* 149, 371-382. 10.1016/j.cell.2012.02.049 22500803 PMC3368265

[JCS219550C38] Maurer S. P., Cade N. I., Bohner G., Gustafsson N., Boutant E. and Surrey T. (2014). EB1 accelerates two conformational transitions important for microtubule maturation and dynamics. *Curr. Biol.* 24, 372-384. 10.1016/j.cub.2013.12.042 24508171 PMC3969257

[JCS219550C39] Melki R., Fievez S. and Carlier M.-F. (1996). Continuous monitoring of Pi release following nucleotide hydrolysis in actin or tubulin assembly using 2-amino-6-mercapto-7-methylpurine ribonucleoside and purine-nucleoside phosphorylase as an enzyme-linked assay. *Biochemistry* 35, 12038-12045. 10.1021/bi961325o 8810908

[JCS219550C40] Montenegro Gouveia S., Leslie K., Kapitein L. C., Buey R. M., Grigoriev I., Wagenbach M., Smal I., Meijering E., Hoogenraad C. C., Wordeman L. et al. (2010). In vitro reconstitution of the functional interplay between MCAK and EB3 at microtubule plus ends. *Curr. Biol.* 20, 1717-1722. 10.1016/j.cub.2010.08.020 20850319

[JCS219550C41] Nakagawa H., Koyama K., Murata Y., Morito M., Akiyama T. and Nakamura Y. (2000). EB3, a novel member of the EB1 family preferentially expressed in the central nervous system, binds to a CNS-specific APC homologue. *Oncogene* 19, 210-216. 10.1038/sj.onc.1203308 10644998

[JCS219550C42] Nakamura S., Grigoriev I., Nogi T., Hamaji T., Cassimeris L. and Mimori-Kiyosue Y. (2012). Dissecting the nanoscale distributions and functions of microtubule-end-binding proteins EB1 and ch-TOG in interphase HeLa cells. *PLoS ONE* 7, e51442 10.1371/journal.pone.0051442 23251535 PMC3520847

[JCS219550C43] Perrone P. A. and Brown P. R. (1984). Ion-pair chromatography of nucleotides. *J. Chromatogr. A* 317, 301-310. 10.1016/S0021-9673(01)91668-1

[JCS219550C44] Preibisch S., Saalfeld S., Schindelin J. and Tomancak P. (2010). Software for bead-based registration of selective plane illumination microscopy data. *Nat. Methods* 7, 418-419. 20508634 10.1038/nmeth0610-418

[JCS219550C45] Rehberg M. and Gräf R. (2002). Dictyostelium EB1 is a genuine centrosomal component required for proper spindle formation. *Mol. Biol. Cell* 13, 2301-2310. 10.1091/mbc.e02-01-0054 12134070 PMC117314

[JCS219550C46] Sandblad L., Busch K. E., Tittmann P., Gross H., Brunner D. and Hoenger A. (2006). The Schizosaccharomyces pombe EB1 homolog Mal3p binds and stabilizes the microtubule lattice seam. *Cell* 127, 1415-1424. 10.1016/j.cell.2006.11.025 17190604

[JCS219550C47] Schroder J. M., Larsen J., Komarova Y., Akhmanova A., Thorsteinsson R. I., Grigoriev I., Manguso R., Christensen S. T., Pedersen S. F., Geimer S. et al. (2011). EB1 and EB3 promote cilia biogenesis by several centrosome-related mechanisms. *J. Cell Sci.* 124, 2539-2551. 10.1242/jcs.085852 21768326 PMC3138699

[JCS219550C48] Seetapun D., Castle B. T., Mcintyre A. J., Tran P. T. and Odde D. J. (2012). Estimating the microtubule GTP cap size in vivo. *Curr. Biol.* 22, 1681-1687. 10.1016/j.cub.2012.06.068 22902755 PMC3461128

[JCS219550C49] Stepanova T., Slemmer J., Hoogenraad C. C., Lansbergen G., Dortland B., De Zeeuw C. I., Grosveld F., Van Cappellen G., Akhmanova A. and Galjart N. (2003). Visualization of microtubule growth in cultured neurons via the use of EB3-GFP (end-binding protein 3-green fluorescent protein). *J. Neurosci.* 23, 2655-2664. 10.1523/JNEUROSCI.23-07-02655.2003 12684451 PMC6742099

[JCS219550C50] Straube A. and Merdes A. (2007). EB3 regulates microtubule dynamics at the cell cortex and is required for myoblast elongation and fusion. *Curr. Biol.* 17, 1318-1325. 10.1016/j.cub.2007.06.058 17658256 PMC1971230

[JCS219550C51] Straube A., Brill M., Oakley B. R., Horio T. and Steinberg G. (2003). Microtubule organization requires cell cycle-dependent nucleation at dispersed cytoplasmic sites: polar and perinuclear microtubule organizing centers in the plant pathogen Ustilago maydis. *Mol. Biol. Cell* 14, 642-657. 10.1091/mbc.e02-08-0513 12589060 PMC149998

[JCS219550C52] Su L. K. and Qi Y. (2001). Characterization of human MAPRE genes and their proteins. *Genomics* 71, 142-149. 10.1006/geno.2000.6428 11161807

[JCS219550C53] Theisen U., Straube E. and Straube A. (2012). Directional persistence of migrating cells requires Kif1C-mediated stabilization of trailing adhesions. *Dev. Cell* 23, 1153-1166. 10.1016/j.devcel.2012.11.005 23237952

[JCS219550C54] Thomas G. E., Bandopadhyay K., Sutradhar S., Renjith M. R., Singh P., Gireesh K. K., Simon S., Badarudeen B., Gupta H., Banerjee M. et al. (2016). EB1 regulates attachment of Ska1 with microtubules by forming extended structures on the microtubule lattice. *Nat. Commun.* 7, 11665 10.1038/ncomms11665 27225956 PMC4894954

[JCS219550C55] Tirnauer J. S., O'toole E., Berrueta L., Bierer B. E. and Pellman D. (1999). Yeast Bim1p promotes the G1-specific dynamics of microtubules. *J. Cell Biol.* 145, 993-1007. 10.1083/jcb.145.5.993 10352017 PMC2133138

[JCS219550C56] Van Der Vaart B., Akhmanova A. and Straube A (2009). Regulation of microtubule dynamic instability. *Biochem. Soc. Trans.* 37, 1007-1013. 10.1042/BST0371007 19754441

[JCS219550C57] Van Der Vaart B., Manatschal C., Grigoriev I., Olieric V., Gouveia S. M., Bjelic S., Demmers J., Vorobjev I., Hoogenraad C. C., Steinmetz M. O. et al. (2011). SLAIN2 links microtubule plus end-tracking proteins and controls microtubule growth in interphase. *J. Cell Biol.* 193, 1083-1099. 10.1083/jcb.201012179 21646404 PMC3115796

[JCS219550C58] Van Haren J., Boudeau J., Schmidt S., Basu S., Liu Z., Lammers D., Demmers J., Benhari J., Grosveld F., Debant A. et al. (2014). Dynamic microtubules catalyze formation of navigator-TRIO complexes to regulate neurite extension. *Curr. Biol.* 24, 1778-1785. 10.1016/j.cub.2014.06.037 25065758

[JCS219550C59] Vitre B., Coquelle F. M., Heichette C., Garnier C., Chrétien D. and Arnal I. (2008). EB1 regulates microtubule dynamics and tubulin sheet closure in vitro. *Nat. Cell Biol.* 10, 415-421. 10.1038/ncb1703 18364701

[JCS219550C60] Von Loeffelholz O., Venables N. A., Drummond D. R., Katsuki M., Cross R. and Moores C. A. (2017). Nucleotide- and Mal3-dependent changes in fission yeast microtubules suggest a structural plasticity view of dynamics. *Nat. Commun.* 8, 2110 10.1038/s41467-017-02241-5 29235477 PMC5727398

[JCS219550C61] Weisbrich A., Honnappa S., Jaussi R., Okhrimenko O., Frey D., Jelesarov I., Akhmanova A. and Steinmetz M. O. (2007). Structure-function relationship of CAP-Gly domains. *Nat. Struct. Mol. Biol.* 14, 959-967. 10.1038/nsmb1291 17828277

[JCS219550C62] Yang C., Wu J., De Heus C., Grigoriev I., Liv N., Yao Y., Smal I., Meijering E., Klumperman J., Qi R. Z. et al. (2017). EB1 and EB3 regulate microtubule minus end organization and Golgi morphology. *J. Cell Biol.* 216, 3179-3198. 10.1083/jcb.201701024 28814570 PMC5626540

[JCS219550C63] Yue J., Xie M., Gou X., Lee P., Schneider M. D. and Wu X (2014). Microtubules regulate focal adhesion dynamics through MAP4K4. *Dev. Cell* 31, 572-585. 10.1016/j.devcel.2014.10.025 25490267 PMC4261153

[JCS219550C64] Zanic M., Stear J. H., Hyman A. A. and Howard J. (2009). EB1 recognizes the nucleotide state of tubulin in the microtubule lattice. *PLoS ONE* 4, e7585 10.1371/journal.pone.0007585 19851462 PMC2761489

[JCS219550C65] Zhang R., Alushin G. M., Brown A. and Nogales E. (2015). Mechanistic origin of microtubule dynamic instability and its modulation by EB proteins. *Cell* 162, 849-859. 10.1016/j.cell.2015.07.012 26234155 PMC4537847

[JCS219550C66] Zhang R., Lafrance B. and Nogales E. (2018). Separating the effects of nucleotide and EB binding on microtubule structure. *Proc. Natl. Acad. Sci. USA* 115, E6191-E6200. 10.1073/pnas.1802637115 29915050 PMC6142192

