# Supplementary material for: Spatial positioning of EB family proteins at microtubule tips involves distinct nucleotide-dependent binding properties
Source: J Cell Sci. 2018 Oct 31;132(4):jcs219550. doi: 10.1242/jcs.219550 (PMC6398475; doi:10.1242/jcs.219550)
Supplement: Supplementary information [file joces-132-219550-s1.pdf]

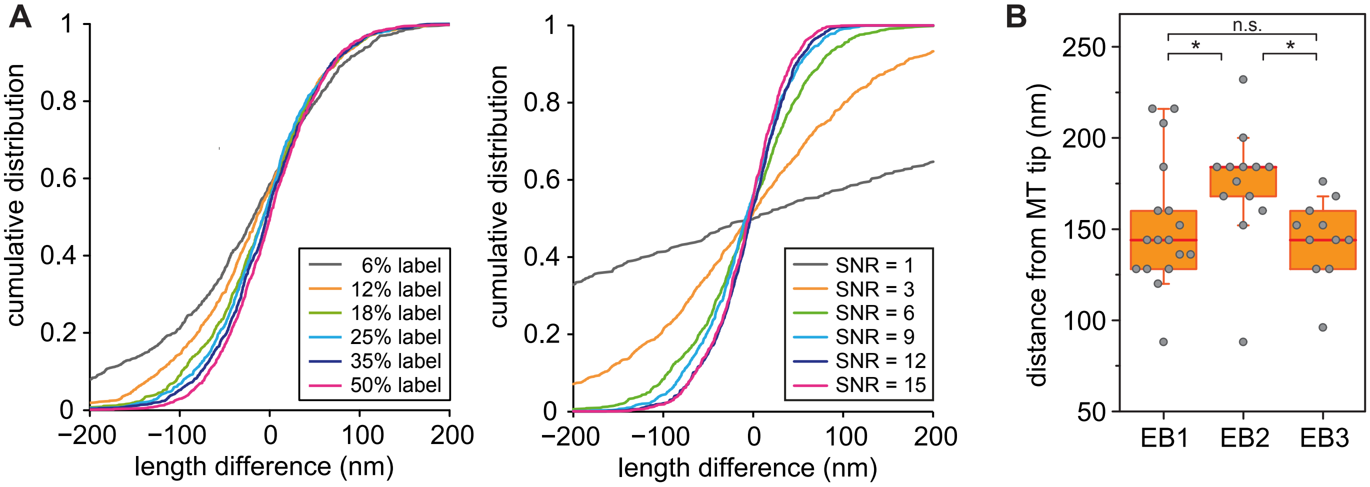

Figure S1: Accuracy of determining microtubule end position. (A) Synthetic images were generated on a 2.5nm subpixel grid, random pixels assigned a label with intensity from Poisson(1.5), convolution with experimental PSF, summing pixels to 80nm grid and addition of real imaging noise. Images stacks of 81 synthetic microtubules growing from 2µm to 4µm at 50nm per frame and shrinking back at the same rate were generated and analysed using our algorithm for finding the microtubule end position in experimental data. Microtubule length was determined from the position of both ends and real microtubule length was subtracted. Data show cumulative distribution of length differences obtained for 10 synthetic image stacks for each condition with varying label density at SNR of 6 and varying SNR at 18% label density. **(B)** Averaged microtubule and EB intensity data as in Figure 3 E-G were calculated separately for each image stack and the distance of the microtubule tip position µ to the position of the maximal value of the averaged EB signal determined. Plot shows the raw data and a box plot with 10, 25, 50, 75, 90 percentiles. n = 12-17 image stacks with 4-15 microtubules each. Statistical significance is shown as * for p<0.05 (Kolmogorov-Smirnov test). Note that the median of these distributions is identical to the peak position of the superaverraged data shown in Fig. 3 E-G

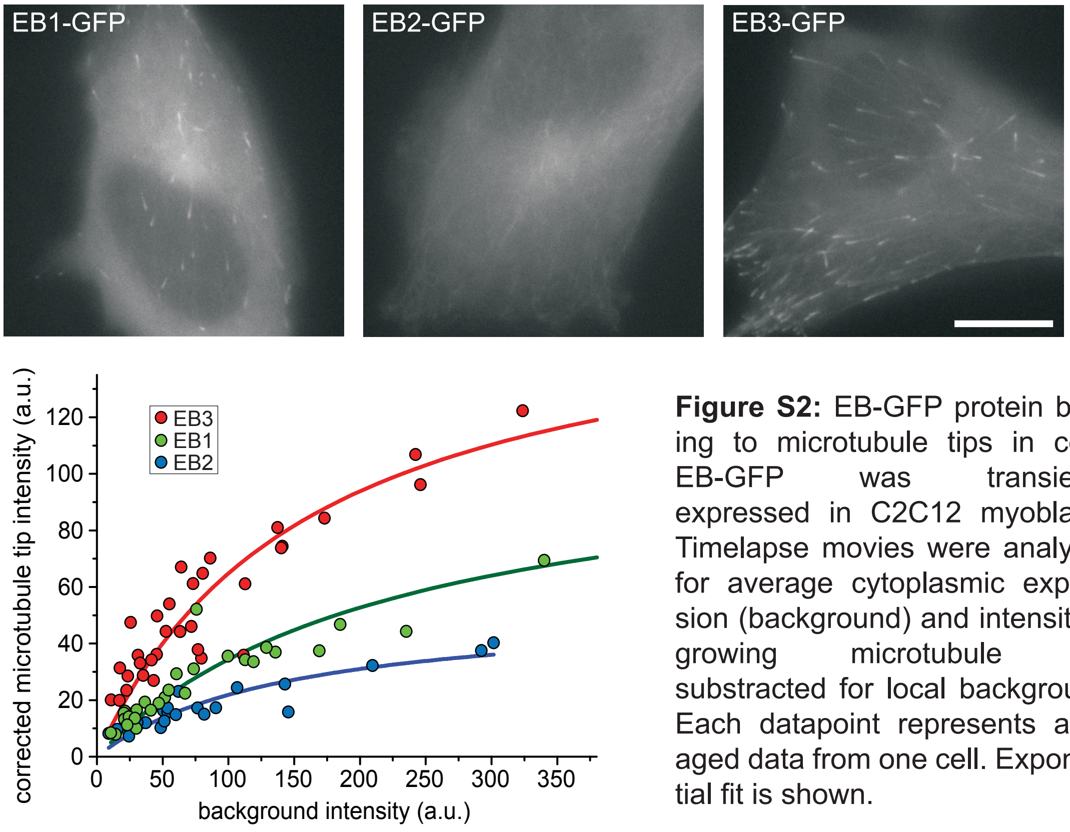

Figure S2: EB-GFP protein binding to microtubule tips in cells. EB-GFP was transiently expressed in C2C12 myoblasts. Timelapse movies were analysed for average cytoplasmic expression (background) and intensity at growing microtubule tips substracted for local background. Each datapoint represents averaged data from one cell. Exponential fit is shown.

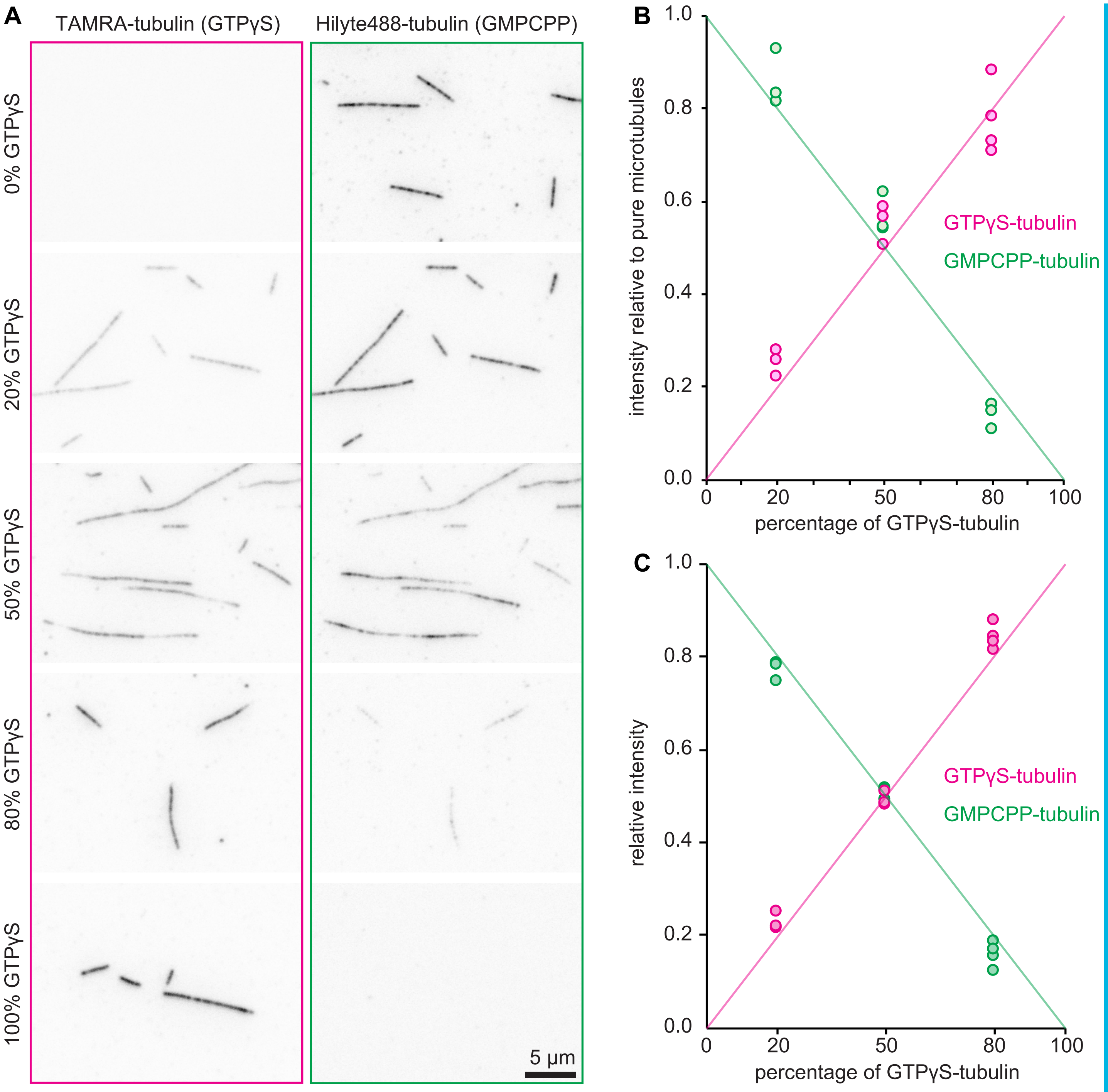

Figure S3: Validation of mixed lattice microtubules. **(A)** Representative images of microtubules co-assembled from tubulin pre-equilibrated with either GMP-CPP or GTPγS in different proportions. Pre-equilibrated tubulin contains 15% labelled tubulin, TAMRA for GTPγS and Hilyte488 for GMPCPP. Both channels are inverted and shown scaled to the same upper and lower intensity values. Scale bar is 5 µm. **(B)** For each experiment, pure and mixed microtubules were imaged in 3 parallel channels of the same coverslip, intensity in both channels was measured for at least 5 fields of view each. Intensity is shown as ratio to pure lattice microtubule. Lines indicate expected distribution if incorporation is proportional. **(C)** Same das as in B, but normalised so that in each chamber the total relative intensity is 1, thus showing relative incorporation. Note that these data suggest equal incorporation in 50% sample and a weak preference for GTPγS/TAMRA-tubulin in the 20% and 80% samples.

Journal of Cell Science • Supplementary information

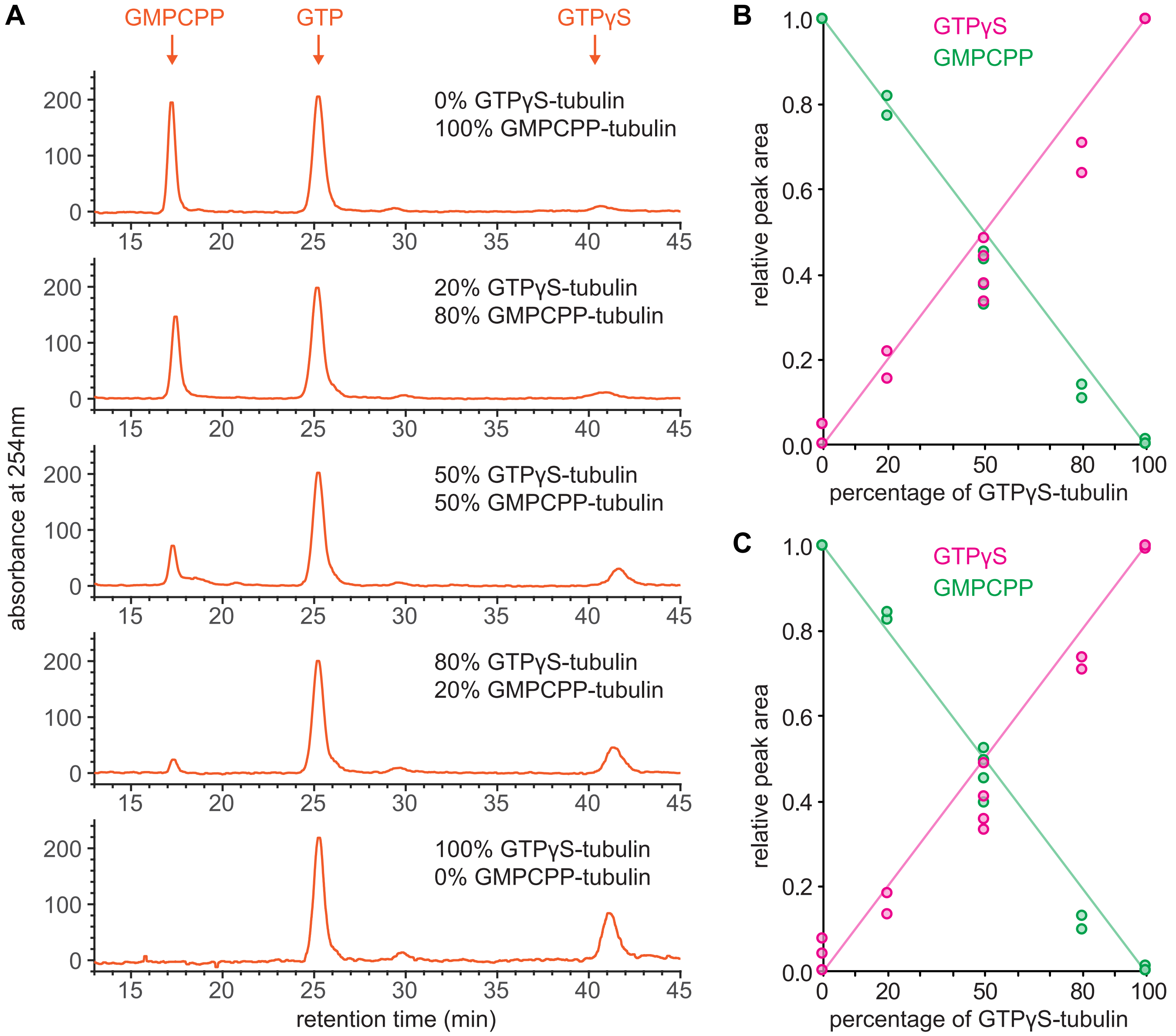

Figure S4: Nucleotide composition of mixed lattice microtubules. (A) Representative HPLC traces of extracted nucleotides from microtubules co-assembled from tubulin pre-equilibrated with either GMP-CPP or GTPγS in different proportions. Note that traces have been normalised to GTP peak intensity to control for differences in efficiency of microtubule formation. **(B-C)** Quantification of peak areas from HPLC traces as those shown in A. Areas under each peak were determined from the raw data and then normalised to either the area of the GTP peak (B) or the sum of GTP, GMPCPP and GTPgS peaks (C) to correct for different amount of microtubules generated. Peak areas are shown relative to pure microtubules analysed on the same day. Lines indicate expected values for equivalent incorporation.

Journal of Cell Science • Supplementary information

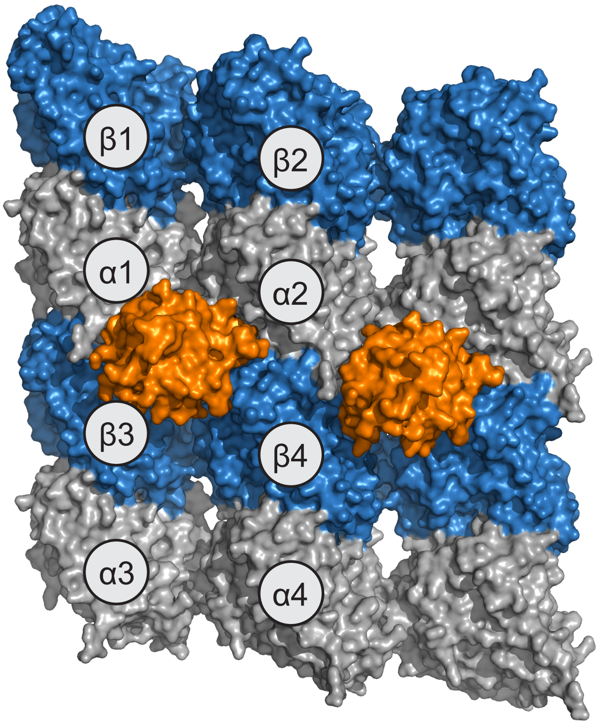

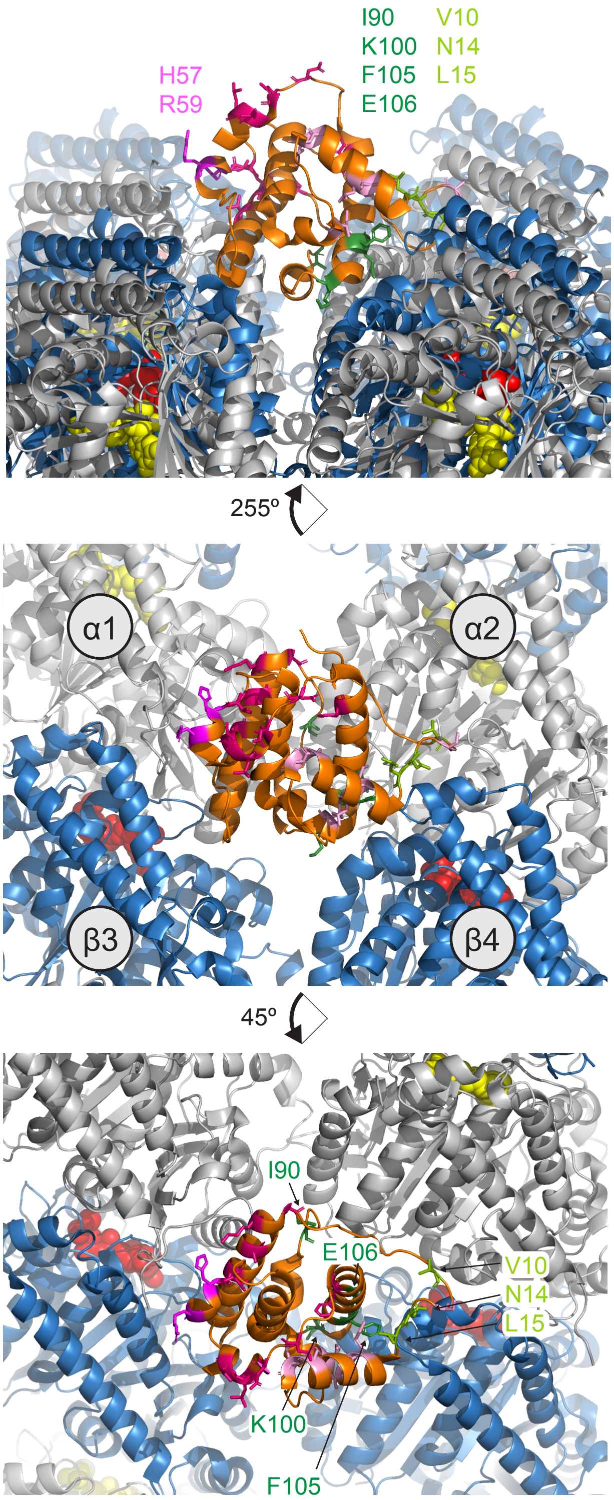

Figure S5: Residues conferring binding specificity to EB2. Human EB3 (orange) bound onto a GTPγS (red) microtubule as in Figure 5B. Amino acids that are substituted in EB2 are shown as sticks with the following colour-code: Those amino acids of EB3 that are present in the EB2-EB3-EB2 chimera are in dark green if within 5Å of a tubulin residue and hot pink if further away from tubulin. Those amino acids that were not in the chimera are shown in rose or in lime green if in proximity of tubulin. The two residues that might make contact with α1 C-terminus in the left protofilament (H57 and R59) and are not conserved between EB3 and EB1 are shown in magenta. All green residues make contact with α2 and β4 in the right protofilament, are conserved between EB1 and EB3, and are thus unique in EB2.

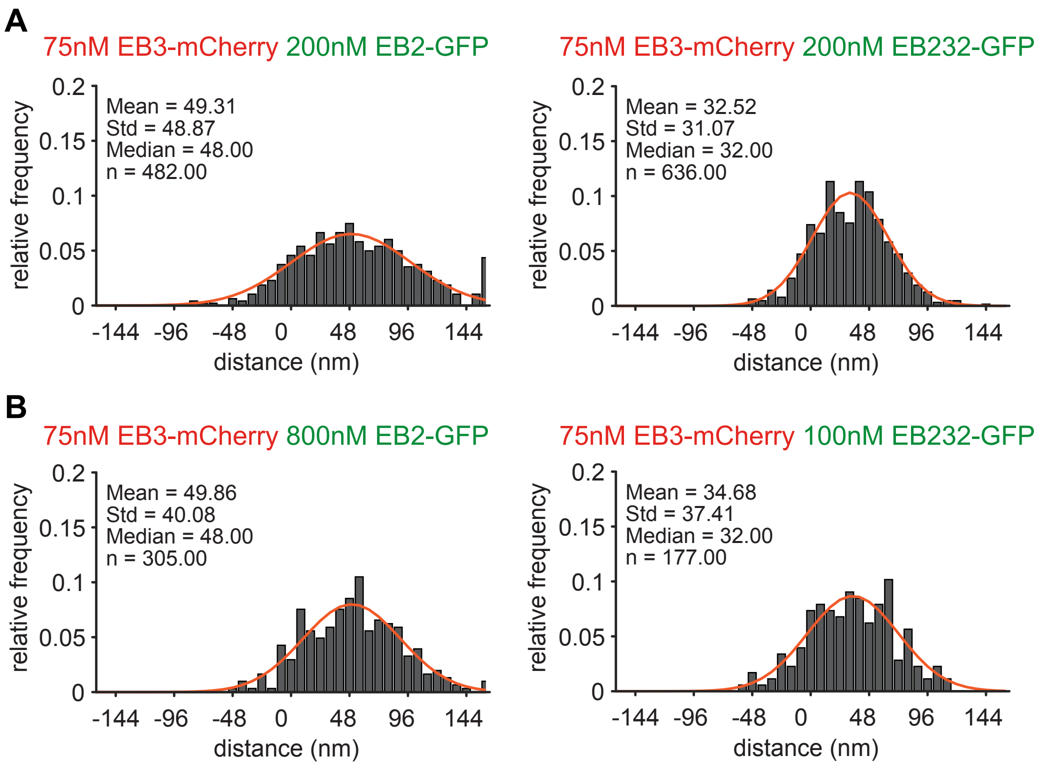

C

EB3-mCherry+	200nM EB2-G	800nM EB2-G	200nM EB232-G	100nM EB232-G
200nM EB2-GFP	1	0.68	6.5e-14	9.6e-06
800nM EB2-GFP	0.68	1	3.5e-12	2.3e-05
200nM EB232-GFP	6.5e-14	3.5e-12	1	0.44
100nM EB232-GFP	9.6e-06	2.3e-05	0.44	1

Figure S6: Peak difference between EB2 and EB3 is independent of EB2 concentration. Intensity profiles were analysed from dual-colour experiments with 75nM EB3-mCherry and different concentrations of EB2-GFP and chimeric EB232-GFP. **(A)** Peak differences are shown for each growth phase analysed for 75nM EB3-mCherry and both EB2 constructs at 200nM - same data as shown in Fig. 8F. **(B)** The experiment was repeated with 100nM EB232-GFP and 800nM EB2-GFP respectively, as at these concentrations tip intensity is comparable of the two proteins (see green curves in Fig. 8 G,H for binding in the presence of EB3-mCherry). **(C)** Table shows p-Values from Mann-Whitney U-tests, significant different results are in green, not different in red. The tests show that there is no difference between the medians of the distributions of the same protein, suggesting that peak differences are independent of EB concentration. However, peak distances of EB232 to EB3 are significantly shorter than for EB2.

Journal of Cell Science • Supplementary information

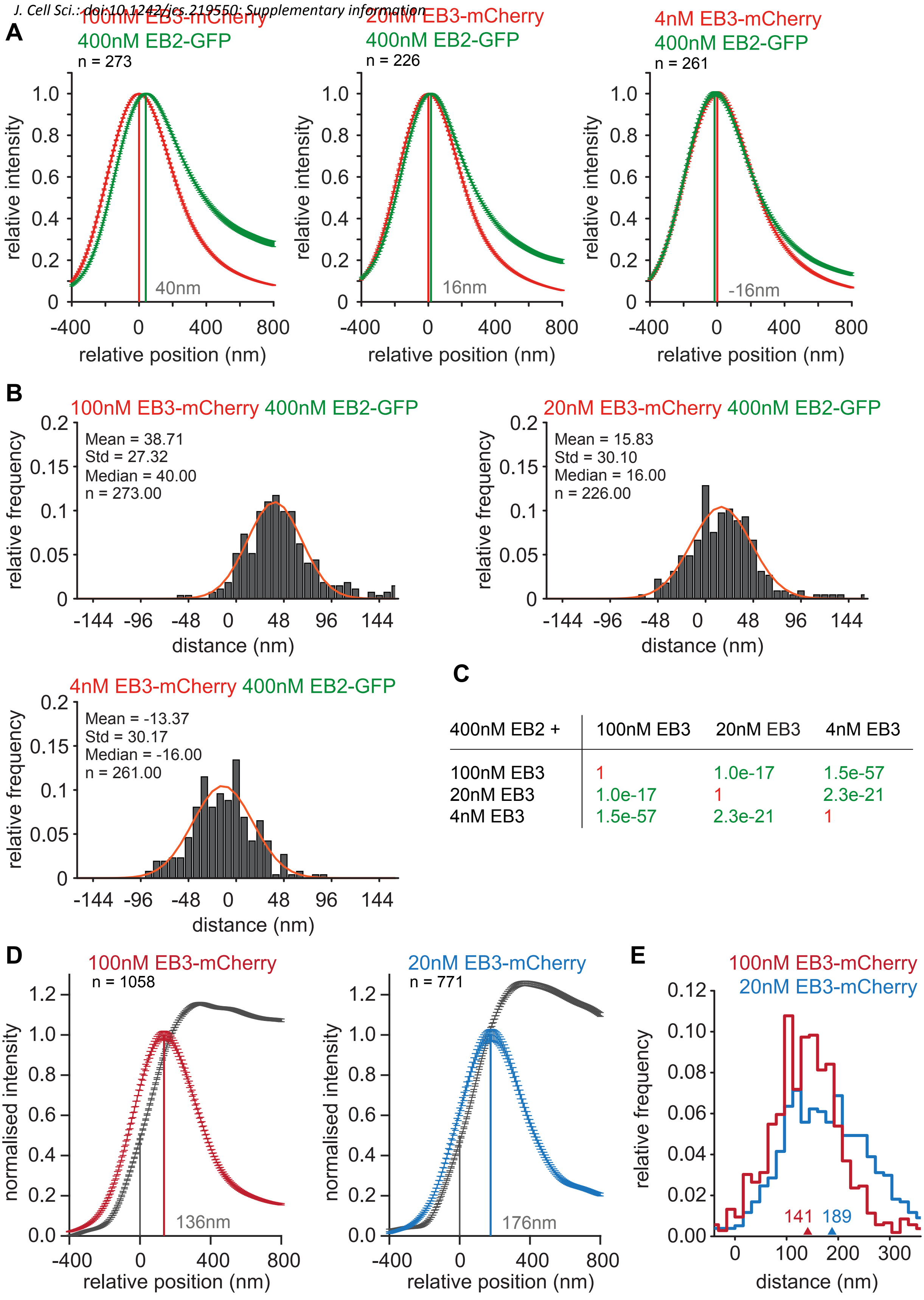

400nM EB2 +	100nM EB3	20nM EB3	4nM EB3
100nM EB3	1	1.0e-17	1.5e-57
20nM EB3	1.0e-17	1	2.3e-21
4nM EB3	1.5e-57	2.3e-21	1

Figure S7: Peak difference between EB2 and EB3 varies with EB3 concentration. (A-C) Intensity profiles were analysed from dual-colour experiments with 400nM EB2-GFP and different concentrations of EB3-mCherry and super-averaged curves are shown in A with grey numbers indicating peak distance. Peak differences are shown for each analysed growth phase in B. Table in C shows p-Values from Mann-Whitney U-tests, significant different results are in green, not different in red. **(D-E)** Position of EB3 comet relative to microtubule tip at two different concentrations of EB3-mCherry. Super-averaged curves are shown in D with peak to microtubule tip distance indicated in grey. Histogram of peak to tip distances shown in E with median indicated with arrowheads. The distributions are significantly different ($p<0.0001$, Kolmogorov-Smirnov test).

Journal of Cell Science • Supplementary information
